# Histone Demethylase JMJD2D Suppresses Influenza A Virus Infection by Promoting RIG-I Expression

**DOI:** 10.3390/biom16040604

**Published:** 2026-04-18

**Authors:** Xiaochun Xia, Jiadi Liang, Hanshi Guo, Fudong Zhang, Junjie Zhang, Chundong Yu, Pingli Mo, Yilin Hong

**Affiliations:** 1 Department of Public Health and Medical Technology, Xiamen Medical College, Xiamen 361023, China; xiaxiaochun@xmmc.edu.cn; 2 State Key Laboratory of Cellular Stress Biology, School of Life Sciences, Xiamen University, Xiamen 361102, China; liangjiadi@stu.xmu.edu.cn (J.L.); 24520211154360@stu.xmu.edu.cn (H.G.); zhangfudong@stu.xmu.edu.cn (F.Z.); 21620250157586@stu.xmu.edu.cn (J.Z.); cdyu@xmu.edu.cn (C.Y.); 3 Xiamen Key Laboratory of Traditional Chinese Medicine Bio-Engineering, School of Pharmacy, Xiamen Medical College, Xiamen 361023, China

**Keywords:** histone demethylase, JMJD2D, influenza A virus, RIG-I

## Abstract

The efficacy of the host antiviral response against Influenza A virus (IAV), a leading cause of global pandemics, hinges upon the rapid recognition of the pathogen and the prompt activation of immune mechanisms. Nevertheless, the epigenetic landscape that orchestrates this antiviral response remains largely elusive. Here, we identify histone demethylase JMJD2D as a critical regulator in defense against IAV infection. A significant upregulation of JMJD2D expression was observed clinically in response to IAV infection, indicating that JMJD2D may play a role in regulating IAV infection. Indeed, JMJD2D-deficient mice exhibit increased susceptibility to IAV, characterized by elevated viral loads, severe lung tissue damage, and reduced survival rates, suggesting that JMJD2D plays an essential role in defense against IAV infection. Consistently, knockdown or pharmacological inhibition of JMJD2D in lung cells suppressed IAV replication and the IAV-triggered innate immune response. Mechanistically, JMJD2D suppressed IAV infection by removing H3K9me3 at the promoter region of retinoic acid inducible gene-I (RIG-I) and cooperating with NF-κB to enhance the expression of RIG-I, a critical sensor for IAV RNA. This study identifies JMJD2D as an epigenetic rheostat that governs RIG-I-mediated antiviral signaling, highlighting its potential as a therapeutic target for mitigating severe IAV infection.

## 1. Introduction

Influenza is an acute respiratory illness caused by infection with viruses of the genus influenza virus, which affects a substantial proportion of the global population annually [1]. Three types of influenza viruses (A, B, and C) of the *Orthomyxoviridae* family infect humans and cause diseases. Among these, influenza A virus (IAV) poses the greatest public health concern, as it is the principal driver of both seasonal outbreaks and global pandemics [2]. At present, H1N1 and H3N2 are the two most common subtypes of prevalent IAV among humans, causing significant public health problems every year [3]. Vaccination remains the most effective strategy for the prevention of influenza [4]. The effectiveness of seasonal influenza vaccines is often diminished by antigenic changes in circulating strains, which allows the virus to escape population immunity [5,6]. Consequently, developing new strategies to combat IAV infection remains a major public health need.

Pattern recognition receptors (PRRs) are sensors of the immune system that serve as a critical line of defense against pathogen invasion. PRRs recognize conserved pathogen-associated molecular patterns (PAMPs), including lipopolysaccharides, nucleic acid motifs, and proteoglycans, and serve as critical initiators of innate immunity [7]. Several families of PRRs are involved in the recognition of influenza viruses, including Toll-like receptors (TLRs) [8], nucleotide-oligomerization-domain-like receptors (NLRs) [9] and retinoic acid inducible gene-I (RIG-I)-like receptors (RLRs) [10]. The RLRs, consisting of RIG-I, LGP2, and MDA5, are cytoplasmic sensors considered the most critical for detecting influenza virus infection [11,12,13]. RIG-I typically recognizes short double-stranded RNA (dsRNA) with 5′-triphosphorylation modification through its C-terminal domain, which is a characteristic of multiple viral genomes [14]. Upon binding viral RNA, RIG-I undergoes conformational changes, exposing its N-terminal CARD domains to engage the mitochondrial antiviral signalling (MAVS) adaptor on mitochondria, thereby triggering signaling cascades that result in the production of interferons and pro-inflammatory cytokines [15]. The activation and downstream signaling of RIG-I are tightly controlled by post-translational modifications such as ubiquitination [16] and ufmylation [15], as well as phase separation [17], to balance effective antiviral defense against excessive inflammation. Previous studies have reported that regulating RIG-I expression contributes to host defense against IAV infection [10,18]. Therefore, precise regulation of the expression and activity of RIG-I is the key to the host defense against viral infection.

Epigenetics, represented by histone modification, plays an important role in the process of viral infection [19]. Histone demethylase JMJD2D (also known as KDM4D) is an epigenetic regulatory factor that activates gene expression by erasing the H3K9me2/3 in the promoter or enhancer regions [20]. JMJD2D emerges in eutherians (placental mammals) through gene family expansion events during the evolution of the JMJD2 family, featuring only the JmjC and JmjN domains [21]. Regarding the function of JMJD2D, JMJD2D is a driving factor for colorectal cancer and liver cancer, by activating the Wnt/β-catenin, Hedgehog [22], HIF1α [23], Notch [24], but suppressing p53 [25] signaling pathways. In terms of inflammation, JMJD2D exerts a positive effect in colitis by activating the Hedgehog pathway to inhibit the apoptosis of colonic epithelial cells and promote their proliferation. Similarly, JMJD2D defends against intestinal bacterial infections by activating the IL-17F/β-defensin axis in colonic epithelial cells [26]. For the inflammatory response, JMJD2D has been revealed to be a positive regulatory factor in the IFN-I response by activating the promoter and enhancer activities [27]. On the contrary, in hepatitis B virus infection, JMJD2D is hijacked to participate in maintaining the stability of HBx and promoting HBV replication [28]. However, the role of JMJD2D in regulation of IAV infection remains unknown.

In this study, we aimed to investigate whether JMJD2D contributes to host defense against IAV and to elucidate the underlying molecular mechanism. We revealed that IAV infection elevated the expression of JMJD2D; JMJD2D-deficient mice were more sensitive to IAV infection; JMJD2D knockdown or inhibition promoted the replication of the IAV and suppressed the innate immune response, while overexpression of JMJD2D did the opposite. These results suggest that JMJD2D plays a critical role in suppressing IAV infection. Mechanistically, JMJD2D erased H3K9me3 on the promoter region of RIG-I and cooperated with NF-κB to promote the expression of RIG-I, which is deeply involved in the host resistance to IAV infection. Collectively, JMJD2D promotes the expression of RIG-I through epigenetic patterns and is indispensable for the host defense against IAV infection.

## 2. Methods and Materials

### 2.1. Cell Lines

The human lung cell line A549, the human embryonic kidney cell line HEK-293T, the canine kidney cell line MDCK, human lung carcinoma H1299 cells and human lung epithelial BEAS-2B cells were procured from the American Type Culture Collection (ATCC) and cultivated in our laboratory. A549 and H1299 cells were cultivated in RPMI 1640 medium, with the addition of 10% fetal bovine serum (FBS) (C04001, VivaCell, Shanghai, China) and 100 U L^−1^ penicillin-streptomycin. HEK-293T, MDCK and BEAS-2B cells were cultivated in Dulbecco’s modified Eagle’s medium (DMEM), with the addition of 10% FBS and 100 U L^−1^ penicillin-streptomycin. The cells were cultivated in an incubator maintained at 37 °C, with a carbon dioxide concentration of 5%.

### 2.2. Knockdown, Overexpression, and Inhibitor Treatment

To generate JMJD2D-knockdown A549 cells, the lentiviral plasmids PLV3-shRNA-JMJD2D-puro-GFP (shJMJD2D) or PLV3-shRNA-puro-GFP (Ctrl) were co-transfected into 293T cells with packaging plasmids PMDL, VSVG, and REV at a ratio of 1:0.5:0.3:0.2. The lentivirus was collected and concentrated, and A549 cells were infected and then selected with puromycin to obtain stable knockdown cells (shJMJD2D-A549). For JMJD2D overexpression, A549 cells were transiently transfected with a pCMV plasmid containing the coding sequence of JMJD2D or with empty pCMV vector using Lipo6000 reagent. The JMJD2D inhibitor 5-c-8HQ was dissolved in DMSO. A549 cells were pretreated with DMSO (control) or 20 μM 5-c-8HQ for 48 h prior to PR8 infection.

### 2.3. Mice

To model human IAV infection, C57BL/6 mice were used as they are a standard influenza model. Mice were housed under specific pathogen-free conditions with a 12 h light/dark cycle, with free access to food and water. The C57BL/6 wild-type (WT) mice and JMJD2D knockout (JMJD2D-KO) mice (C57BL/6 background) were previously obtained from our laboratory. Male mice aged 6–8 weeks, obtained from the Xiamen University Animal Center, were selected for the experimental protocol. The animal study protocol was approved by the Institutional Review Board of Xiamen University Laboratory Animal Center (protocol code No. XMULAC20230046, approved on 14 March 2023). Isoflurane anaesthesia was used during intranasal inoculation to minimize distress. No unexpected adverse events occurred; expected weight loss following IAV infection was observed. Humane endpoints were set at >25% body weight loss or moribund state, with daily monitoring.

### 2.4. Virus

The mouse-adapted IAV (H1N1, PR8) utilised in this study was kindly provided by Professor Chen Yixin (School of Life Sciences, Xiamen University). Previous studies have confirmed the lethality of this virus in mice [29]. The viral titers were determined by infecting MDCK cells and expressed as 50% tissue culture infectious dose per milliliter (TCID50) according to the method of Reed and Muench [30]. The amplification of the H1N1 virus was facilitated by MDCK cells. Tosylsulfonyl phenylalanyl chloromethyl ketone (TPCK)-treated trypsin was utilised in all experiments involving cell infection. All experiments involving live virus were conducted in the BSL-2 biosafety facility at Xiamen University.

### 2.5. Viral Infection

For the cells, A549 cells were first washed with phosphate-buffered saline (PBS) and infected with a multiplicity of infection (MOI) of 0.1 of the IAV strain PR8. The infection medium contained 0.5 mg/mL TPCK-treated trypsin and 0.5% bovine serum albumin (BSA). After one hour, the cells were transferred to fresh infection medium and then cultivated in an incubation chamber set to 37 °C until the specified time point.

For the murine model, the mice were anaesthetised with isoflurane and administered either PBS or PR8 (50 μL per mouse, 50 LD_50_) via intranasal instillation. Body weight was measured on a daily basis following infection. At the conclusion of the experiment, the mice were euthanised and the lung tissue was collected for subsequent histopathological analysis. Right lung lobules were then homogenised, after which RNA and protein extracts were prepared for subsequent assays. The survival of the mice was recorded in independent experiments.

### 2.6. Real-Time Quantitative PCR (RT-qPCR)

Total RNA was extracted and purified from tissues and cells using Trizol (AG21102, AGbio, Hangzhou, China). The All-In-One 5× RT MasterMix (G592, abm, Richmond, BC, Canada) was utilised in the synthesis of cDNA from total RNA. Gene expression changes were detected using 2× Universal SYBR Green Fast qPCR Mix (RK21203, ABclonal, Wuhan, China) on the ROCHE LightCycler 480II. Gene expression levels were normalised using β-actin and calculated using comparative threshold (Ct) cycle method (2^−ΔΔCt).

The RT-qPCR primer sequences utilised are as follows:

h-β-actin, Forward 5′-TAGCACCATGAAGATCAAGAT-3′, Reverse 5′-CCGATCCACACAGAGTACTT-3′;

h-JMJD2D, Forward 5′-ACGCTATGACCTGTGGAAAC-3′, Reverse 5′-TCTCCTGGGTAACTGGACTT-3′;

h-IFNβ, Forward 5′-CTTGGATTCCTACAAAGAAGCAGC-3′, Reverse 5′-TCCTCCTTCTGGAACTGCTGCA-3′;

h-ISG56, Forward 5′-GCCTTGCTGAAGTGTGGAGGAA-3′, Reverse 5′-ATCCAGGCGATAGGCAGAGATC-3′;

h-RIG-I, Forward 5′-CACCTCAGTTGCTGATGAAGGC-3′, Reverse 5′-GTCAGAAGGAAGCACTTGCTACC-3′;

IAV-M, Forward 5′-TGGACAAAGCAGTTAAACTGTATA-3′, Reverse 5′-CTTCAGTGGTCACAGTCCCCATCC-3′;

IAV-NP, Forward 5′-GGCCGTCATGGTGGCGAAT-3′, Reverse 5′-CTCAATATGAGTGCAGACCGTGCT-3′.

### 2.7. Western Blot

Cells and tissues were lysed using RIPA buffer (containing protease and phosphatase inhibitors). Protein concentration was determined by the BCA assay. Equivalent amounts of protein were separated by SDS-PAGE gel electrophoresis and transferred to PVDF membranes for antibody incubation and visualization.

The following antibodies were used in Western blot:

anti-JMJD2D (Cat# ab93694, Abcam, Cambridge, UK), anti-GAPDH (Cat# AC033, ABclonal), anti-IAV NP (Cat# 99797T, CST, Danvers, MA, USA), anti-RIG-I (Cat# 20566-1-AP, Proteintech, Rosemont, IL, USA), anti-NF-κB (Cat# 8242S, CST).

### 2.8. Luciferase Reporter Analysis

Cells were co-transfected with reporter plasmid, Renilla luciferase plasmid (internal control), target plasmid. The collection of cell supernatants was performed using lysis buffer. After adding the fluorescent substrate, the fluorescence intensity was measured 24 h after transfection. Luciferase activity was normalised using sea urchin luciferase activity.

### 2.9. Co-Immunoprecipitation (Co-IP) Assay

Total cellular protein was extracted using a lysis buffer containing a protease inhibitor, with 5% of the volume reserved as input. The remaining protein lysate was mixed with pre-washed Protein A/G (washed three times with lysis buffer) and the designated antibody. The mixture was then incubated overnight at 4 °C with gentle rotation. The coprecipitates were washed with buffer, separated by SDS-PAGE gel electrophoresis, and subsequently subjected to Western blot using specific antibodies.

### 2.10. Chromatin Immunoprecipitation (ChIP) Assay

ChIP experiments were performed as previously described. In brief, the cells were fixed and cross-linked using 1% formaldehyde solution, after which glycine termination was performed. The chromatin was then immunoprecipitated using specific antibodies and Protein A/G. The ChIP DNAs were then extracted, purified and analysed by RT-qPCR using RIG-I promoter primers. The RIG-I ChIP primer used in this study was listed as follows:

h-ChIP-RIG-I-promoter, Forward 5′-TCCTAGGGGTCCTCTCCGTT-3′, Reverse 5′-GGCCTCTGCTTGCAGCTAGC-3′.

### 2.11. RNA-Sequencing Analysis

The A549 cells that had been knocked down for JMJD2D, along with their control cells, were collected and sent to BGI (Shenzhen, China) for extraction, sample preparation, sequencing and analysis.

### 2.12. AI Tool(s)

In this study, DeepSeek AI (DeepSeek-V3) was utilized exclusively for the purposes of language editing and grammatical correction. The objective of this utilization was to enhance the clarity, readability, coherence, and presentation of the manuscript. All AI-generated content was subjected to a rigorous review process, involving thorough verification, critical evaluation and editing by the respective authors.

### 2.13. Statistical Analysis

Statistical analysis was performed using GraphPad Prism 9. Quantitative data were expressed as the mean ± standard error of the mean (SEM), with experiments repeated at least three times. The significance of the data was assessed using Student’s *t*-tests. *p* value < 0.05 was considered statistically significant.

## 3. Results

### 3.1. JMJD2D Is Induced in Response to IAV Infection

**Figure 1:**
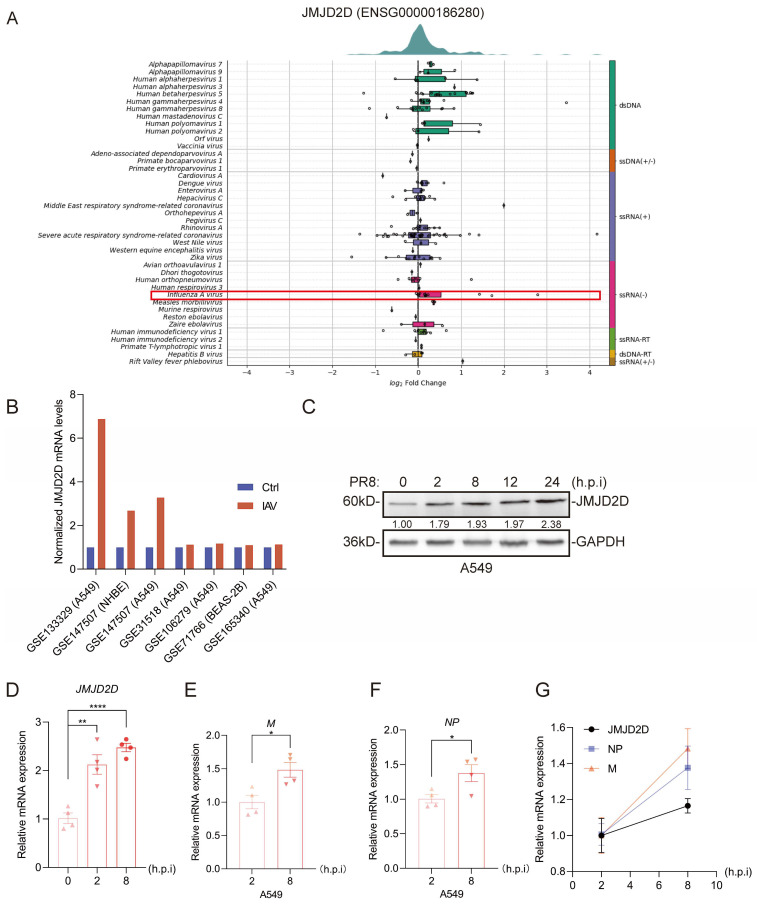
The level of JMJD2D is increased during IAV infection. (**A**) Virus Expression Database analysis reveals altered JMJD2D expression in response to viral infection. The red frame highlights the altered JMJD2D expression in response to IAV. (**B**) According to the Virus Expression Database analysis, JMJD2D expression is upregulated in lung-associated cells upon IAV infection. (**C**,**D**) Western blot (**C**) and RT-qPCR (**D**) analysis of JMJD2D expression in A549 cells infected with PR8 (MOI = 0.1, *n* = 3 per group). (**E**,**F**) In A549 cells, the levels of M (**E**) and NP (**F**) at 8 h.p.i were significantly higher than those at 2 h.p.i. (**G**) Correlation analysis revealed a positive correlation between JMJD2D expression and the mRNA levels of viral NP and M. Results are representative of one of three independent experiments. Data are presented as means ± SEM. * *p* < 0.05, ** *p* < 0.01, **** *p* < 0.0001, based on Student’s *t* test. Original images of (**C**) can be found in Supplementary Materials.

Given that epigenetic regulators often modulate host responses to pathogens [19], we hypothesized that JMJD2D expression might be dynamically regulated during IAV infection. To test this, we first examined its expression levels in the Virus Expression Database. We observed that infection with multiple distinct viruses upregulated JMJD2D expression, suggesting a potential role for JMJD2D in antiviral defense (Figure 1A). IAV infection induced a marked increase in JMJD2D expression in various human pulmonary cell lines (Figure 1B). We next assessed JMJD2D expression in A549 cells following IAV infection. Notably, viral infection significantly enhanced JMJD2D protein (Figure 1C) and mRMA (Figure 1D) expression. In addition, the RNA levels of M and NP were examined at various time points post-infection with PR8. The results demonstrated that the levels of M (Figure 1E) and NP (Figure 1F) increased significantly at 8 h.p.i. compared to 2 h.p.i. We observed a positive correlation between JMJD2D levels and the expression of NP and M (Figure 1G). These results suggest that JMJD2D is a potential host factor involved in defense against IAV infection.

### 3.2. JMJD2D Deficiency Enhances IAV Susceptibility in Mice

**Figure 2:**
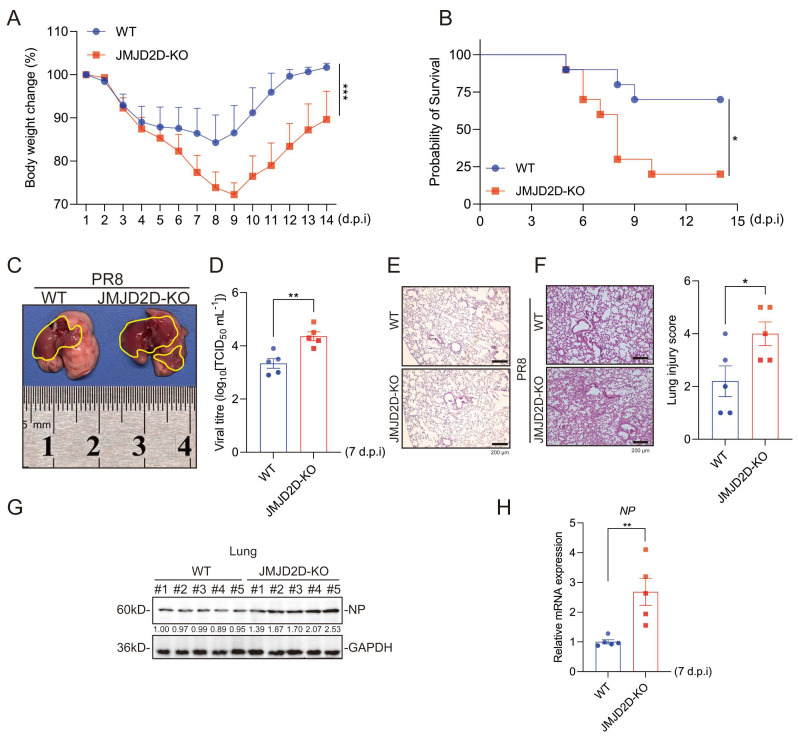
JMJD2D deficiency aggravates IAV-induced pathogenesis. (**A**) Body weight loss was monitored daily for 14 days following IAV infection in WT and JMJD2D-KO mice (*n* = 9 per group). (**B**) Survival rates of WT and JMJD2D-KO mice (*n* = 10 per group) following lethal IAV infection. (**C**) JMJD2D-KO mice developed more severe lung necrosis than WT mice at 7 days post-infection (d.p.i) with IAV (*n* = 5 per group). The yellow area indicates lung injury and necrosis induced by IAV infection. (**D**) Following PR8 infection, increased viral titers were observed in the lungs of JMJD2D-KO mice compared to WT mice (*n* = 5 per group). (**E**) H&E-stained lung tissues from uninfected WT and JMJD2D-KO mice (*n* = 3 per group). (**F**) Representative H&E-stained lung sections from WT and JMJD2D-KO mice at 7 d.p.i. Lung injury was scored on a scale of 1–5 by three investigators blinded to the experimental groups (*n* = 5 per group). (**G**) Western blot analysis of PR8 NP protein levels in lung lysates from WT and JMJD2D-KO mice at 7 d.p.i (*n* = 5 per group). (**H**) RT-qPCR analysis of viral NP mRNA levels in the lungs of WT and JMJD2D-KO mice at 7 d.p.i (*n* = 5 per group). Body weight measurement and survival analysis were each performed in duplicate, and similar results were obtained. Other findings are representative of one of three independent experiments. Data are presented as means ± SEM. * *p* < 0.05, ** *p* < 0.01, *** *p* < 0.001, based on Student’s *t* test. Original images of (**G**) can be found in Supplementary Materials.

To investigate the role of JMJD2D in IAV infection in vivo, 7- to 8-week-old WT and JMJD2D-KO mice were intranasally infected with the PR8 influenza virus strain. Upon PR8 infection, JMJD2D-KO mice experienced more severe body weight loss and delayed weight regain relative to WT mice (Figure 2A). Additionally, PR8-infected JMJD2D-KO mice exhibited significantly lower survival rates compared to WT mice (Figure 2B). Given that the lung is the primary target organ for IAV [10], we examined pulmonary pathological damage following PR8 infection. As expected, gross pathological analysis revealed that PR8-infected JMJD2D-KO mice had more extensive tissue damage compared to WT mice (Figure 2C). We measured viral titers by TCID50 assay using lung homogenates from infected mice. As observed previously, JMJD2D-KO mice had higher viral titers in lung tissues than WT controls (Figure 2D). As a control, we examined H&E-stained lung sections from uninfected WT and JMJD2D-KO mice (Figure 2E). Next, histopathological analysis showed that the lung tissues of JMJD2D-KO mice had more severe inflammation and a higher damage score (Figure 2F). Moreover, higher levels of viral NP were detected in the lung tissue of JMJD2D-KO mice than in WT mice (Figure 2G,H). Collectively, these results suggest that JMJD2D is a critical host factor for protection against IAV infection in vivo.

### 3.3. JMJD2D Protects Against IAV Infection in Lung Cells

**Figure 3:**
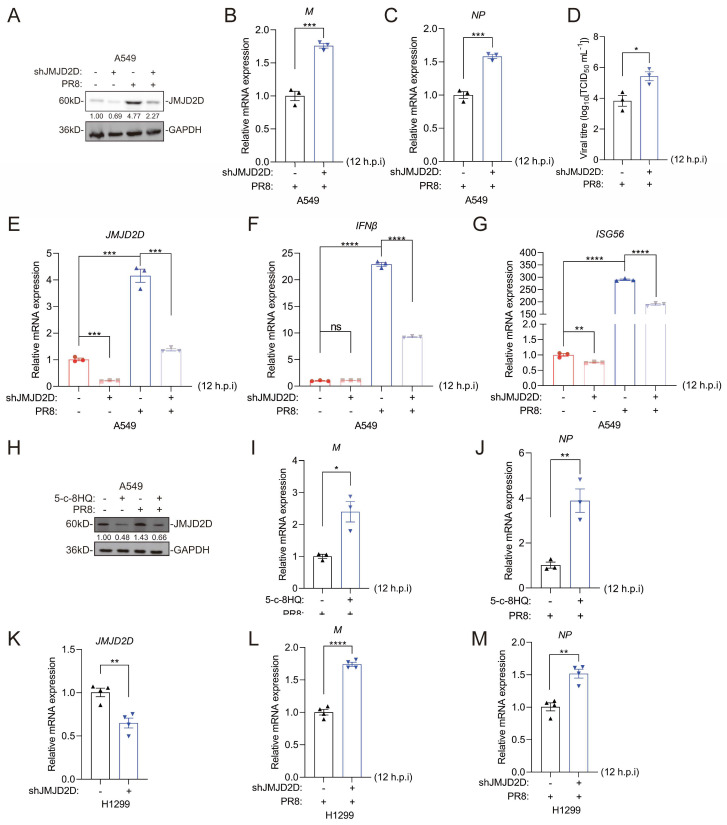
JMJD2D deficiency promotes PR8 infection in A549 cells. (**A**–**G**) Knockdown of JMJD2D increases viral replication and suppresses antiviral gene expression (viral MOI = 0.1). Knockdown efficiency was confirmed by Western blot (**A**) and RT-qPCR (**E**). Viral M (**B**) and NP (**C**) mRNA levels were assessed by RT-qPCR. PR8 viral titers were increased in JMJD2D-knockdown A549 cells (**D**). The expression of antiviral genes IFNβ (**F**) and ISG56 (**G**) was measured by RT-qPCR (*n* = 3 per group). (**H**–**J**) Pharmacological inhibition of JMJD2D enhances PR8 replication in A549 cells. Cells were pretreated with 20 μM 5-c-8HQ for 48 h, followed by infection with PR8 (MOI = 0.1). Samples were collected at 12 h post-infection. JMJD2D protein levels were analyzed by Western blot (**H**), and mRNA expression levels of viral M (**I**) and NP (**J**) were measured by RT-qPCR (*n* = 3 per group). (**K**–**M**) JMJD2D knockdown (**K**) upregulates the level of M (**L**) and NP (**M**) in PR8-infected H1299 cells (*n* = 4 per group). All results are representative of three independent experiments. Data are presented as means ± SEM. * *p* < 0.05, ** *p* < 0.01, *** *p* < 0.001, **** *p* < 0.0001, based on Student’s *t* test. Original images of (**A**,**H**) can be found in Supplementary Materials.

**Figure 4:**
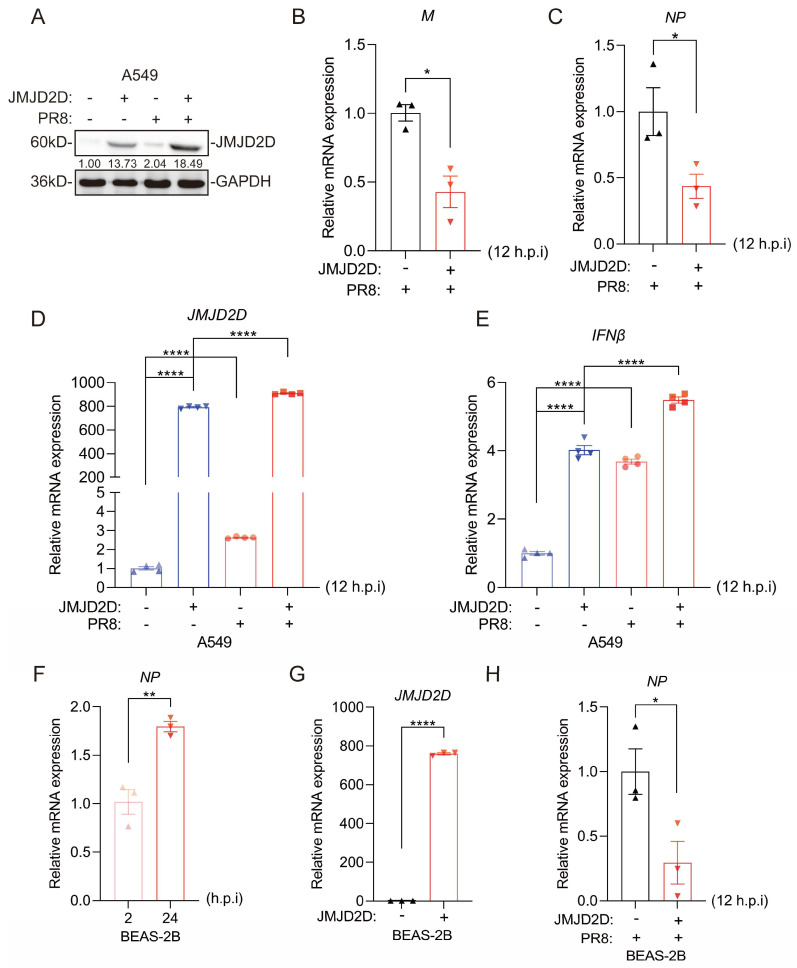
Overexpression of JMJD2D reduces viral load of IAV in A549 cells. (**A**) Western blot was performed to detect JMJD2D protein expression in A549 cells following JMJD2D overexpression and PR8 infection. (**B**–**E**) The mRNA expression levels of viral M (**B**) and NP (**C**) (*n* = 3 per group), as well as JMJD2D (**D**) and IFNβ (**E**) (*n* = 4 per group), were assessed by RT-qPCR in A549 cells overexpressing JMJD2D following PR8 infection. (**F**) PR8 infection increases NP levels in BEAS-2B cells at 24 h.p.i compared to 2 h.p.i (*n* = 3 per group). (**G**,**H**) Overexpression of JMJD2D (**G**) reduces NP (**H**) levels in BEAS-2B cells. All results are representative of three independent experiments. Data are presented as means ± SEM. * *p* < 0.05, ** *p* < 0.01, **** *p* < 0.0001, based on Student’s *t* test. Original images of (**A**) can be found in Supplementary Materials.

Given that JMJD2D is essential for host defense against IAV infection in vivo, we sought to determine whether JMJD2D plays an important role in IAV infection in vitro. To investigate the function of endogenous JMJD2D during IAV infection, JMJD2D-knockdown A549 cells were infected with PR8 virus (Figure 3A). JMJD2D-knockdown A549 cells infected with IAV exhibited higher mRNA levels of viral M and NP at 12 h.p.i (Figure 3B,C). Knocking down JMJD2D in A549 cells raised viral titers (Figure 3D). Type I interferons (IFN-I) and interferon-stimulated genes (ISGs) play a critical role in host resistance to IAV infection [31]. Consistent with this, JMJD2D knockdown significantly reduced the expression of IFNβ and ISG56 in PR8-infected A549 cells. (Figure 3E–G). The JMJD2D inhibitor 5-c-8HQ abrogated PR8-induced JMJD2D protein expression in A549 cells (Figure 3H). This inhibition correlated with enhanced viral M and NP gene expression following PR8 infection (Figure 3I,J). Similar results were seen in H1299 cells, where JMJD2D knockdown raised M and NP levels (Figure 3K–M). Additionally, we overexpressed JMJD2D in A549 cells (Figure 4A), and subsequently infected them with PR8 virus. Similarly, overexpression of JMJD2D downregulated the expression of PR8 viral M and NP (Figure 4B,C), while upregulating IFNβ in A549 cells (Figure 4D,E). In BEAS-2B cells, NP levels go up at 24 h.p.i compared to 2 h.p.i (Figure 4F). Overexpression of JMJD2D brings NP levels down (Figure 4G,H). Collectively, these results demonstrate that JMJD2D functions as a positive regulator of innate antiviral immunity, suppressing IAV infection in cultured cells.

### 3.4. JMJD2D Upregulates the Expression of RIG-I

**Figure 5:**
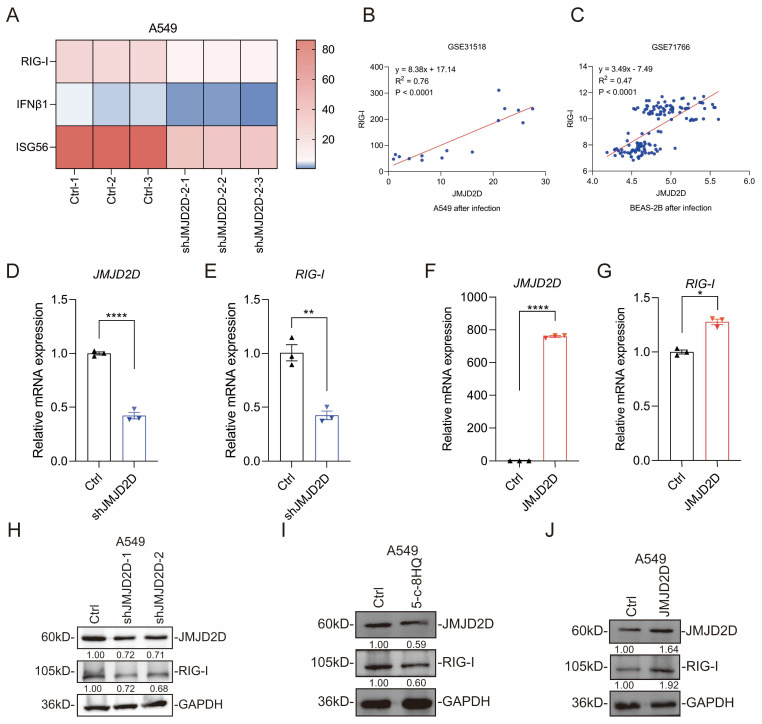
JMJD2D upregulates the expression of RIG-I. (**A**) Heatmap analysis of RNA-seq data revealed that JMJD2D knockdown reduced the mRNA expression of RIGI and IFNβ1. (**B**,**C**) Analysis of GEO datasets GSE31518 and GSE71766 revealed a positive correlation between JMJD2D and RIGI expression in human lung cell lines A549 (**B**) and BEAS-2B (**C**) following IAV infection. (**D**,**E**) RT-qPCR analysis confirmed that JMJD2D knockdown (**D**) suppresses RIG-I (**E**) mRNA levels in A549 cells (*n* = 3 per group). (**F**,**G**) RT-qPCR analysis confirmed that JMJD2D overexpression (**F**) increased RIG-I (**G**) mRNA levels in A549 cells (*n* = 3 per group). (**H**) JMJD2D knockdown inhibits RIG-I expression at the protein level in A549 cells. (**I**) RIG-I protein expression is inhibited in A549 cells following treatment with 20 μM 5-c-8HQ for 48 h. (**J**) JMJD2D overexpression increases RIG-I protein expression. All results are representative of three independent experiments. Data are presented as means ± SEM. * *p* < 0.05, ** *p* < 0.01, **** *p* < 0.0001, based on Student’s *t* test. Original images of (**H**–**J**) can be found in Supplementary Materials.

RIG-I receptor senses cytoplasmic viral RNA and activates IFN-I and downstream antiviral immune responses [32]. RNA-seq profiling of JMJD2D-knockdown A549 cells revealed a significant downregulation of RIG-I and downstream antiviral effectors, such as IFNβ1 and ISG56 (Figure 5A). In addition, public data analysis showed that the expression levels of JMJD2D and RIG-I were positively correlated after IAV infection of A549 (GSE31518) (Figure 5B) and BEAS-2B (GSE71766) (Figure 5C) cells. At the transcriptional level, knockdown of JMJD2D reduced the mRNA level of RIG-I (Figure 5D,E), while overexpression of JMJD2D increased the mRNA level of RIG-I (Figure 5F,G). Consistently, knockdown or pharmacological inhibition (5-c-8HQ) of JMJD2D downregulated RIG-I protein expression (Figure 5H,I), while overexpression of JMJD2D increased the protein level of RIG-I (Figure 5J). These results imply that JMJD2D suppresses IAV infection by enhancing RIG-I expression.

### 3.5. JMJD2D Promotes RIG-I-Mediated RNA Stress Response

**Figure 6:**
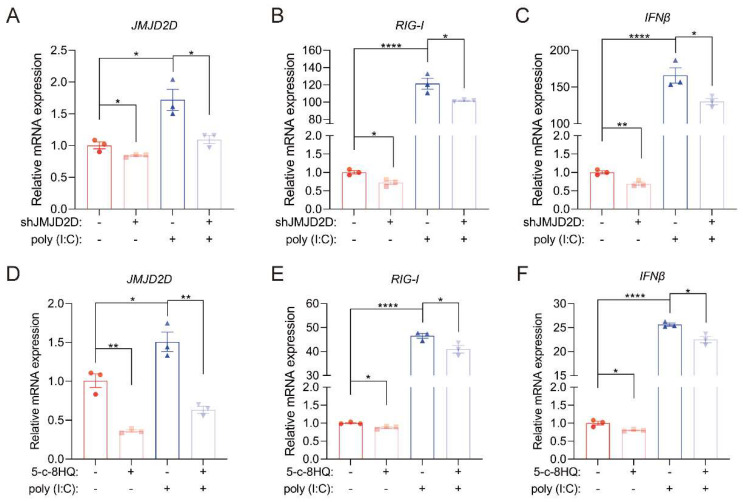
JMJD2D promotes RIG-I-mediated RNA stress response. (**A**–**C**) JMJD2D (**A**), RIG-I (**B**), and IFNβ (**C**) expression were assessed in A549 cells subjected to JMJD2D knockdown followed by transfection with 1 μg poly (I:C) for 24 h (*n* = 3 per group). (**D**–**F**) The mRNA expression levels of JMJD2D (**D**), RIG-I (**E**), and IFNβ (**F**) were assessed by RT-qPCR. Cells were pretreated with 20 μM 5-c-8HQ for 48 h, followed by transfection with 1 μg poly (I:C) for 24 h to mimic PR8 infection (*n* = 3 per group). All results are representative of three independent experiments. Data are presented as means ± SEM. * *p* < 0.05, ** *p* < 0.01, **** *p* < 0.0001, based on Student’s *t* test.

Given that RIG-I recognizes viral RNA and triggers antiviral innate immune responses, we sought to determine whether JMJD2D participates in this process. Knockdown of JMJD2D in A549 cells suppressed the expression of RIG-I and IFNβ upon stimulation with poly (I:C), a viral RNA mimic used to simulate viral infection (Figure 6A–C). Pharmacological inhibition of JMJD2D with 5-c-8HQ attenuated poly (I:C)-induced RIG-I and IFNβ expression in A549 cells (Figure 6D–F). These results demonstrate that JMJD2D is essential for initiating antiviral innate immune responses by enhancing RIG-1 expression to further induce the expression of IFNβ.

### 3.6. JMJD2D Cooperates with NF-κB to Promote the Expression of RIG-I

JMJD2D, as a transcriptional co-activator, unbinds dense heterochromatin by removing histone H3K9me2/3 modification, and recruits the transcription factors to activate the transcription of target genes. Previous studies have reported the involvement of JMJD family proteins in NF-κB-mediated inflammatory responses [33], and epigenetic regulation has been shown to modulate NF-κB-dependent immune functions [34].

**Figure 7:**
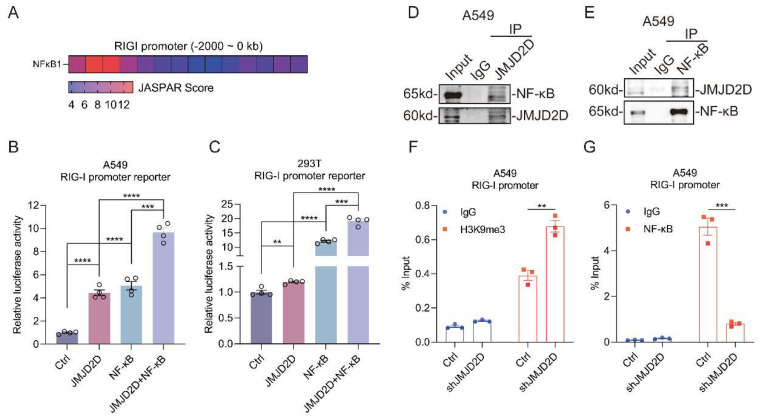
JMJD2D cooperates with NF-κB to transcriptionally activate RIG-I expression. (**A**) JASPAR prediction revealed potential NF-κB binding sites in the RIG-I promoter region. (**B**,**C**) Luciferase reporter assays revealed that JMJD2D cooperates with NF-κB to enhance RIG-I promoter activity in A549 cells (**B**) and HEK293T cells (**C**) (*n* = 4 per group). (**D**,**E**) Endogenous JMJD2D interacts with NF-κB in A549 cells. Immunoprecipitates obtained with anti-JMJD2D antibody were immunoblotted with anti-NF-κB antibody (**D**). Immunoprecipitations obtained with anti-NF-κB antibody were immunoblotted with anti-JMJD2D antibody (**E**). (**F**,**G**) ChIP-qPCR analysis revealed that JMJD2D knockdown increased H3K9me3 (**F**) enrichment at the RIG-I promoter while reducing NF-κB (**G**) recruitment to this region in A549 cells (*n* = 3 per group). All results are representative of three independent experiments. Data are presented as means ± SEM. ** *p* < 0.01, *** *p* < 0.001, **** *p* < 0.0001, based on Student’s *t* test. Original images of (**D**,**E**) can be found in Supplementary Materials.

To investigate whether NF-κB directly regulates RIG-I transcription, we analyzed the RIG-I promoter using the JASPAR database. This analysis uncovered multiple putative NF-κB binding sites upstream of the RIG-I gene (Figure 7A). Therefore, we hypothesized that JMJD2D may act as a transcriptional co-activator in coordination with NF-κB to regulate the transcription of RIG-I. To verify this hypothesis, we performed luciferase reporter assays in HEK293T and A549 cells. Exogenous expression of JMJD2D and NF-κB significantly activated the luciferase activity of the RIG-I promoter (Figure 7B,C). More importantly, co-expression of JMJD2D and NF-κB cooperatively elevated the promoter activity of RIG-I, indicating that JMJD2D cooperates with NF-κB to enhance RIG-I transcription.

Furthermore, we conducted Co-IP analysis to examine whether JMJD2D physically interacts with NF-κB. As expected, JMJD2D interacted with NF-κB in A549 cells (Figure 7D,E). Again, we performed ChIP assay to determine whether JMJD2D promotes the binding of NF-κB by reducing the H3K9me3 modification in the RIG-I promoter region. Indeed, knockdown of JMJD2D increased the histone H3K9me3 modification in the RIG-I promoter region and reduced the binding of NF-κB to RIG-I promoter region (Figure 7F,G). In conclusion, these results indicate that JMJD2D elevates the binding of NF-κB by removing the H3K9me3 to enhance the transcriptional activity of RIG-I.

## 4. Discussion

In this study, we revealed that JMJD2D is a crucial regulatory factor of the host defense against IAV infection. IAV infection upregulates JMJD2D expression, and loss of JMJD2D increases host susceptibility to IAV accompanied by impaired innate antiviral immunity in both cell culture and mouse models. In contrast, overexpression of JMJD2D inhibits the replication of the IAV and activates innate antiviral immunity. Mechanistically, JMJD2D promotes RIG-I transcription by erasing H3K9me3 from its promoter and acting as a co-activator for NF-κB. This upregulation of RIG-I is key to sensing viral RNA and initiating downstream antiviral responses. This study highlights the essential role of JMJD2D in innate immunity and expands the current view of JMJD2D-mediated epigenetic regulation in immune defense. Moreover, it suggests a novel approach to counter IAV infection and points to JMJD2D as a promising therapeutic target.

We observed an elevated expression of JMJD2D in IAV infection, which prompted us to elucidate the role of JMJD2D in defending against IAV. JMJD2D-KO mice exhibited high viral load, severe lung tissue damage and poorer survival rate, indicating that JMJD2D is a protective factor for the host against IAV. The increase in the expression level of JMJD2D may precisely be the correct decision made by the host to resist IAV. Previous studies have found that TNFα-mediated NF-κB signaling promotes the expression of JMJD2D in colonic epithelial cells, playing a protective role in colitis [22]. Coincidentally, the infection of IAV also triggers the NF-κb [35], MAPK [36], IRFs [37] inflammatory signaling pathways. More importantly, in this study, JMJD2D acts as a co-activator to cooperate with NF-κB, and previous research has revealed that JMJD2D is also a target gene of NF-κB. This seems to suggest that the elevated expression of JMJD2D in IAV infection is a cascading amplification effect. In fact, the elevated expression of JMJD2D in viral and bacterial infections is a widespread phenomenon [22,27,29], although the role of JMJD2D may be contrary. In addition, members of the JMJD2 family are widely involved in the viral infection process. For instance, in herpesvirus infections, JMJD2 members are recruited by the cell co-activator HCF-1, which removes the inhibitory histone marker H3K9me3 from the viral IE gene promoter, creating an open chromatin environment for transcriptional activation [38]. The struggle between the host and the virus reshaped by this epigenetic regulatory factor seems to be a widespread regulatory paradigm.

The defense against IAV mainly relies on a rapid and effective host immune response. Among them, IFN-I are the vanguards of the host antiviral response, and viral infection induces a strong expression of IFN-I [39]. IFN-I promotes the formation of ISGF3 by binding to receptors and activating the JAK-STATs signaling pathway. ISGF3 entering the nucleus activates the expression of numerous ISGs [40]. Our research reveals that JMJD2D is indispensable for the activation of host IFN-I in IAV infection. Previous epigenetic studies have demonstrated that JMJD2D is a positive regulator of IFN-I responses by erasing H3K9me3 on the promoter and enhancer of the corresponding IFN-I gene [27]. In addition to innate immunity, the activation of adaptive immunity requires IFN-I. After IAV infection, IFN-I promotes the differentiation of naive CD8^+^ T cells into cytotoxic T lymphocytes [41]. Therefore, these findings suggest that JMJD2D may coordinate the rapid response of innate immunity and the ordered activation of adaptive immunity by regulating the expression of IFN-I. RIG-I is regarded as the most sensitive sensor for identifying cytoplasmic viral dsRNA [13]. We revealed that JMJD2D cooperates with NF-κB to promote the expression of RIG-I in an epigenetic regulatory pattern. However, after IAV infection, elevated expression of RIG-I constitutes just one facet of the underlying mechanism. What is more important is the activation of RIG-I activity [42]. For example, TRIM25-mediated Lys63(K63)-linked ubiquitination is necessary for RIG-I activation [16,43]. Subsequently, a series of RIG-I ubiquitination sites and ubiquitin ligases were identified [12,44,45]. In addition, the activity of RIG-I is also regulated by phosphorylation modification. Casein kinase 2 (CK2) phosphorylates the Thr770, Ser854 and Ser855 sites of RIG-I, which may be necessary to maintain RIG-I in an autorepressed state [46]. Coincidentally, in HBV infection, JMJD2D inhibits the ubiquitin-proteasome degradation pathway mediated by E3 ubiquitin ligase TRIM14 through interaction with HBx, significantly enhancing the stability of HBx protein and preventing the rapid degradation of HBx [28]. This implies that JMJD2D may be involved in the activity regulation of RIG-I mediated by post-translational modifications.

While previous studies have clearly established the role of JMJD2D in tumorigenesis, inflammation regulation, and DNA damage repair, our research uncovers its positive role in antiviral infection. Given the complexity and multidimensionality of host antiviral responses during IAV infection, JMJD2D may exert broad effects across different stages of IAV infection and various cell types. Furthermore, the plasticity of JMJD2D as an epigenetic regulator endows it with considerable druggable potential. Despite these important insights, several critical questions remain to be addressed: (1) Insufficient evidence exists to confirm whether JMJD2D regulates RIG-I expression entirely through its demethylase activity. (2) The increased susceptibility of systemic JMJD2D-KO mice cannot be fully explained by the loss of JMJD2D function in lung cells, as the contributions of numerous immune cell subsets cannot be ruled out. (3) Although our study establishes that JMJD2D contributes to host defense against IAV in the early stage through RIG-I-mediated transcriptional regulation, its potential role in the late phase of infection, which is often marked by immune dysregulation, warrants further investigation. Addressing these remaining questions and elucidating the underlying mechanistic details will be the primary objectives of our subsequent investigations. The current study focused on JMJD2D’s function against H1N1 (PR8), a double-stranded RNA virus. What JMJD2D does during infection with other influenza subtypes, such as H3N2, is still unclear and will be part of our next study.

## 5. Conclusions

In conclusion, our research indicates that IAV infection-mediated JMJD2D positively regulates the host antiviral response by inhibiting viral replication and enhancing the innate immune response. Mechanically, JMJD2D, as a co-activator, cooperates with NF-κB to promote the expression of RIG-I by erecting the inhibitory histone modification H3K9me3 in the promoter region, which is the double-stranded RNA receptor of the virus. This study reveals the important mechanism of JMJD2D in the antiviral response and expands our understanding of epigenetic regulation in virus recognition and clearance, which may contribute to the development of novel antiviral targets.

## Data Availability

All data generated or analyzed during this study are included in this article/Supplementary Material. Further inquiries can be directed to the corresponding author.

## Supplementary Materials

The following supporting information can be downloaded at: https://www.mdpi.com/article/10.3390/biom16040604/s1, Original images of Figure 1C, Figure 2G, Figure 3A,H, Figure 4A, Figure 5H–J and Figure 7D,E can be found in supplementary materials.

## Abbreviations

The following abbreviations are used in this manuscript:

ATCC	American Type Culture Collection
BSA	bovine serum albumin
ChIP	Chromatin Immunoprecipitation
CK2	Casein kinase 2
Co-IP	Co-Immunoprecipitation
Ct	comparative threshold
DMEM	Dulbecco’s modified Eagle’s medium
DsRNA	double-stranded RNA
FBS	fetal bovine serum
IAV	influenza A virus
IFN-I	Type I interferons
ISGs	interferon-stimulated genes
JMJD2D-KO	JMJD2D-deficient
K63	Lys63
MAVS	mitochondrial antiviral signalling
MOI	multiplicity of infection
NLRs	nucleotide-oligomerization-domain-like receptors
PAMPs	pathogen-associated molecular patterns
PBS	phosphate-buffered saline
PRRs	Pattern recognition receptors
RIG-I	retinoic acid inducible gene-I
RLRs	RIG-I-like receptors
SEM	standard error of the mean
TCID50	50% tissue culture infectious dose per milliliter
TLRs	Toll-like receptors
TPCK	Tosylsulfonyl phenylalanyl chloromethyl ketone
WT	wild-type
