# Supplementary figures and images for: Histone Demethylase JMJD2D Suppresses Influenza A Virus Infection by Promoting RIG-I Expression
Source: Biomolecules. 2026 Apr 18;16(4):604. doi: 10.3390/biom16040604 (PMC13115551; doi:10.3390/biom16040604)

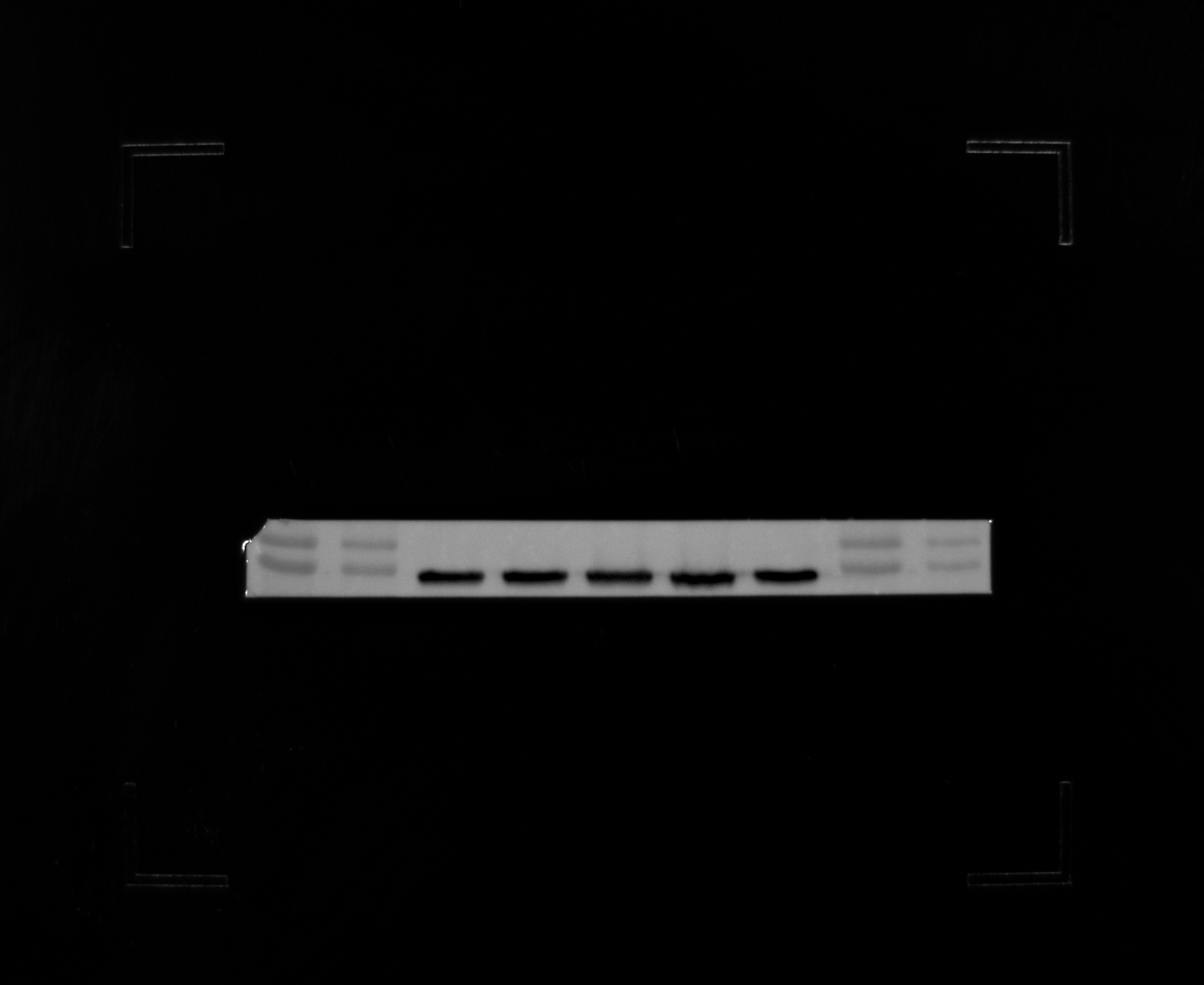

Supplement: Supplementary file 1 [file biomolecules-16-00604-s001.zip › Fig 1C-GAPDH.tif]

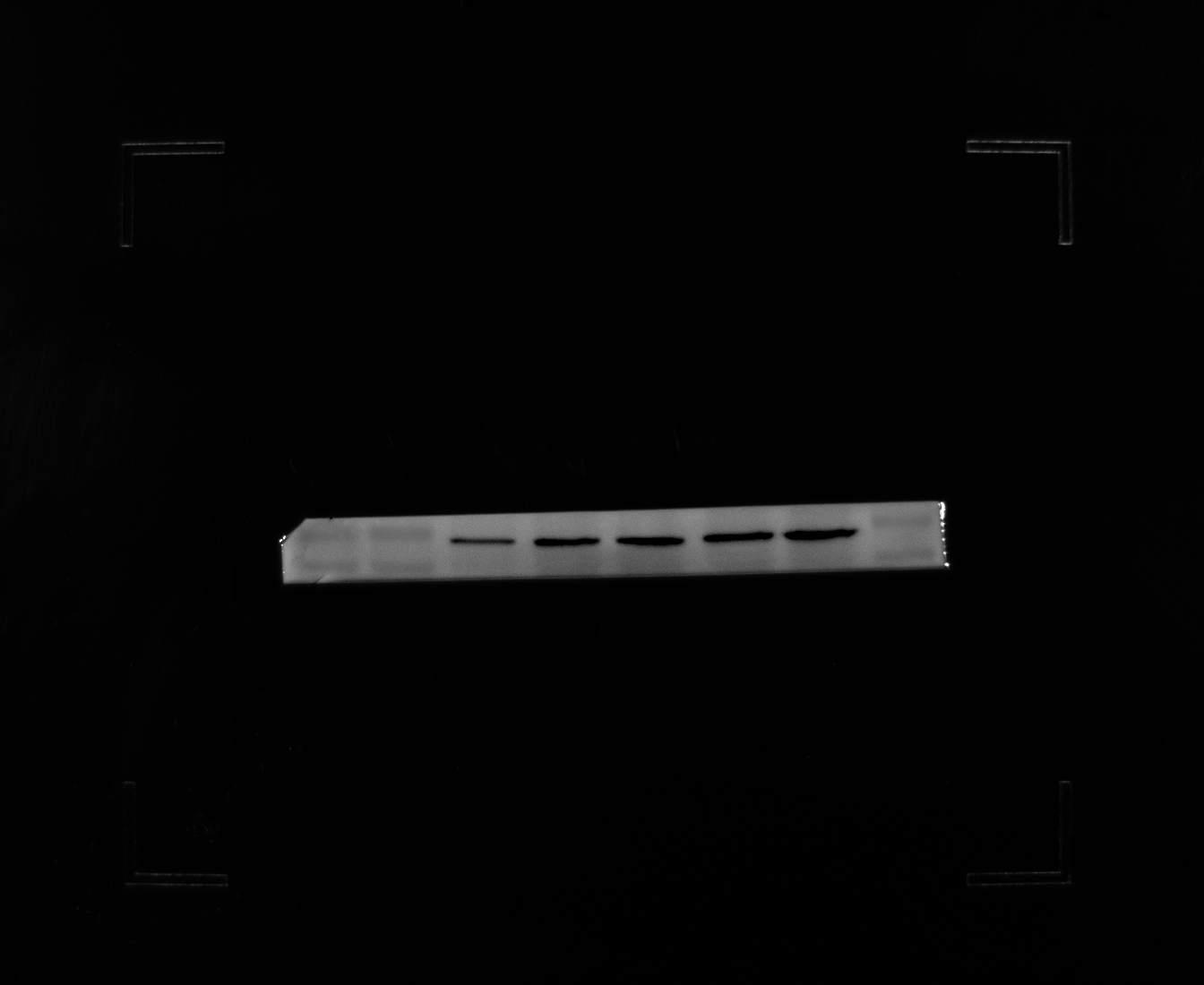

Supplement: Supplementary file 1 [file biomolecules-16-00604-s001.zip › Fig 1C-JMJD2D.tif]

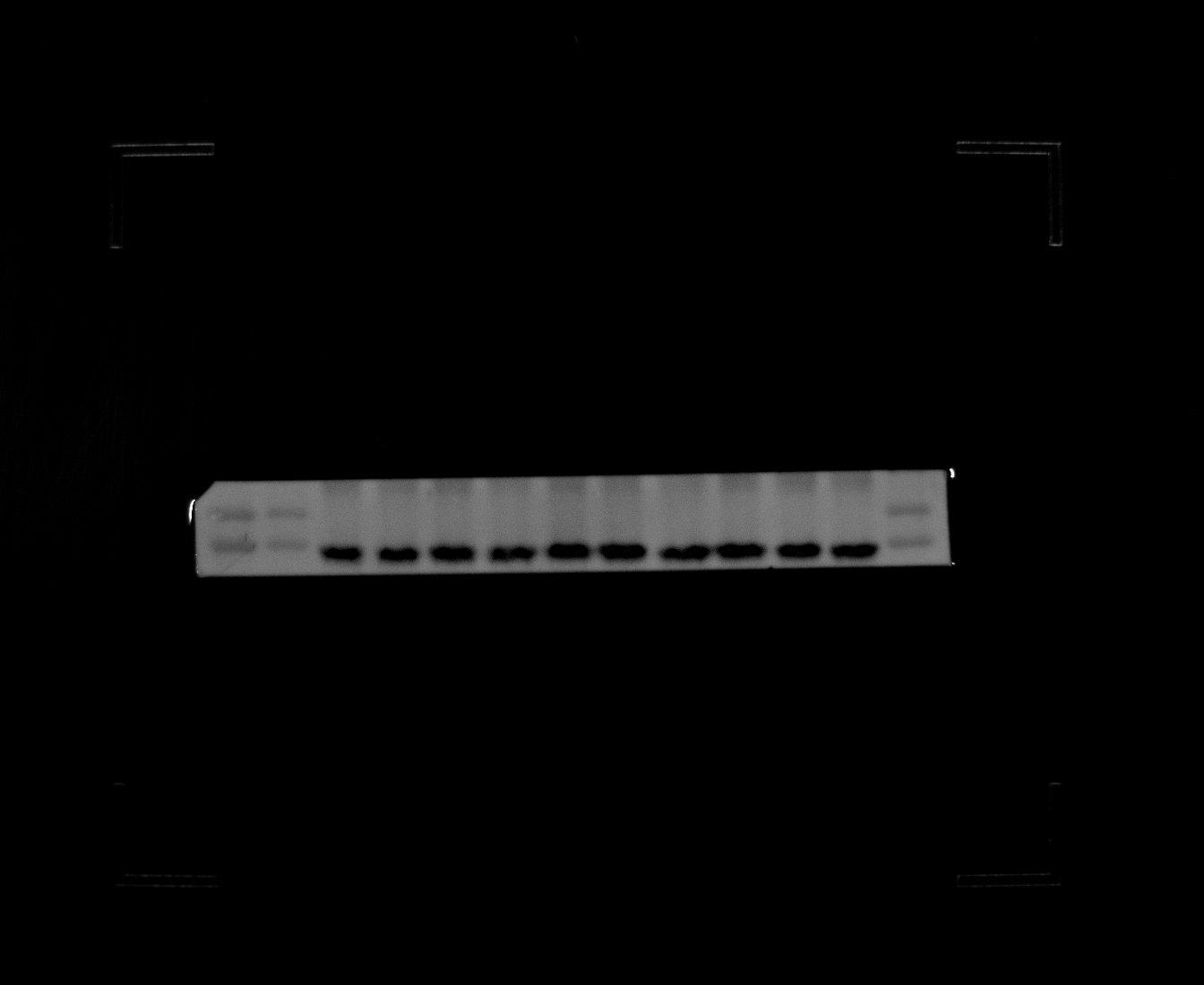

Supplement: Supplementary file 1 [file biomolecules-16-00604-s001.zip › Fig 2G-GAPDH.tif]

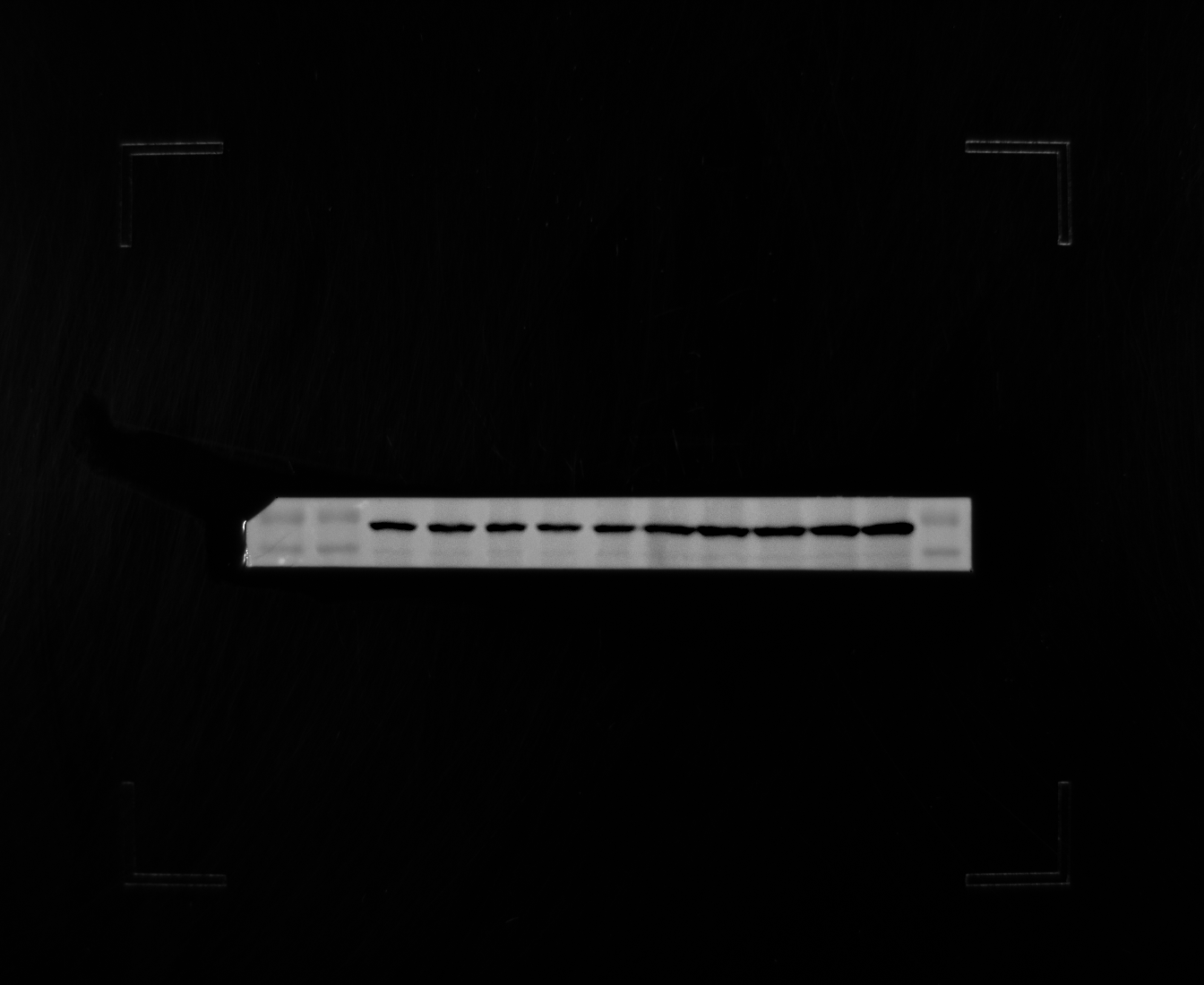

Supplement: Supplementary file 1 [file biomolecules-16-00604-s001.zip › Fig 2G-NP.tif]

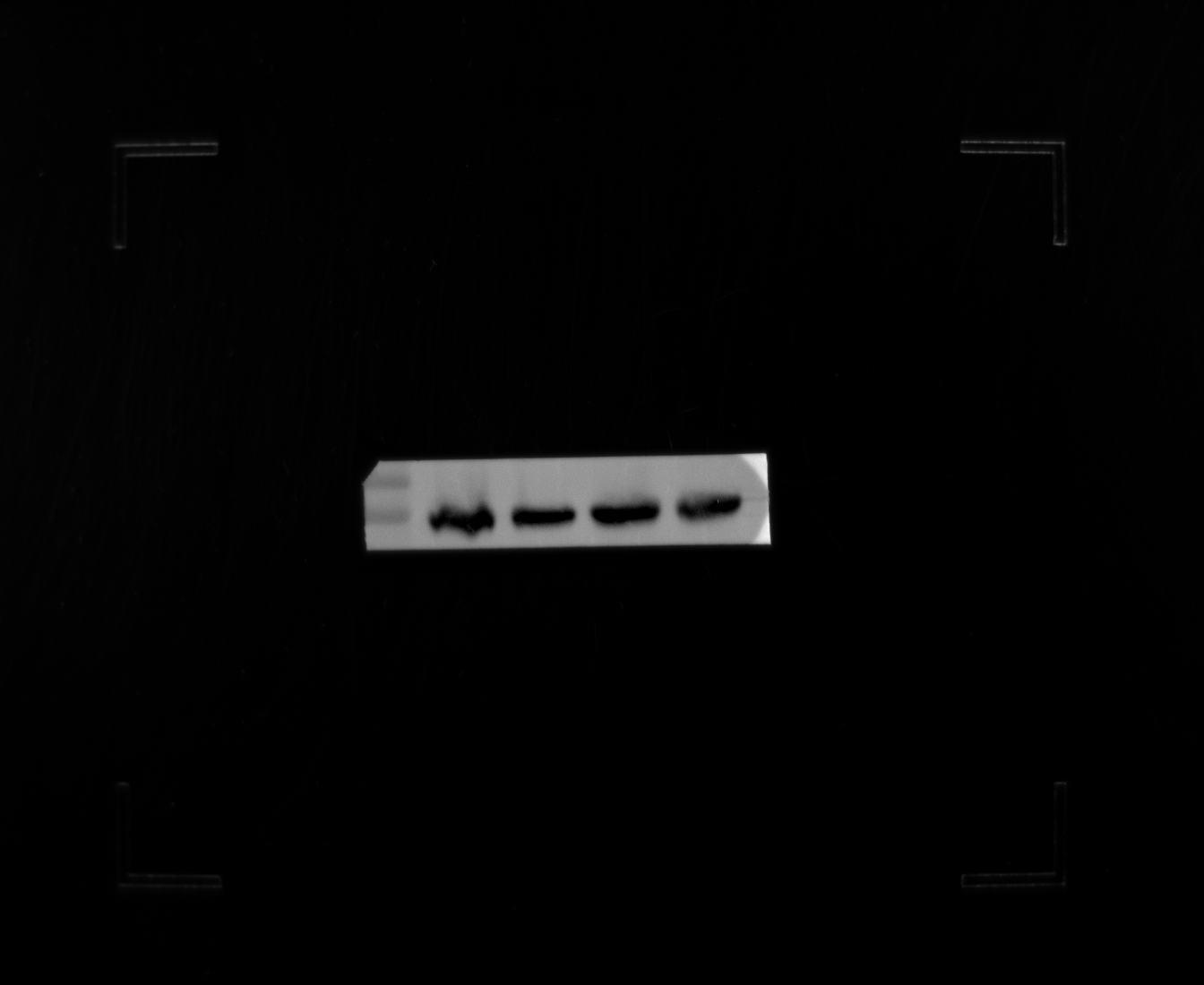

Supplement: Supplementary file 1 [file biomolecules-16-00604-s001.zip › Fig 3A-GAPDH.tif]

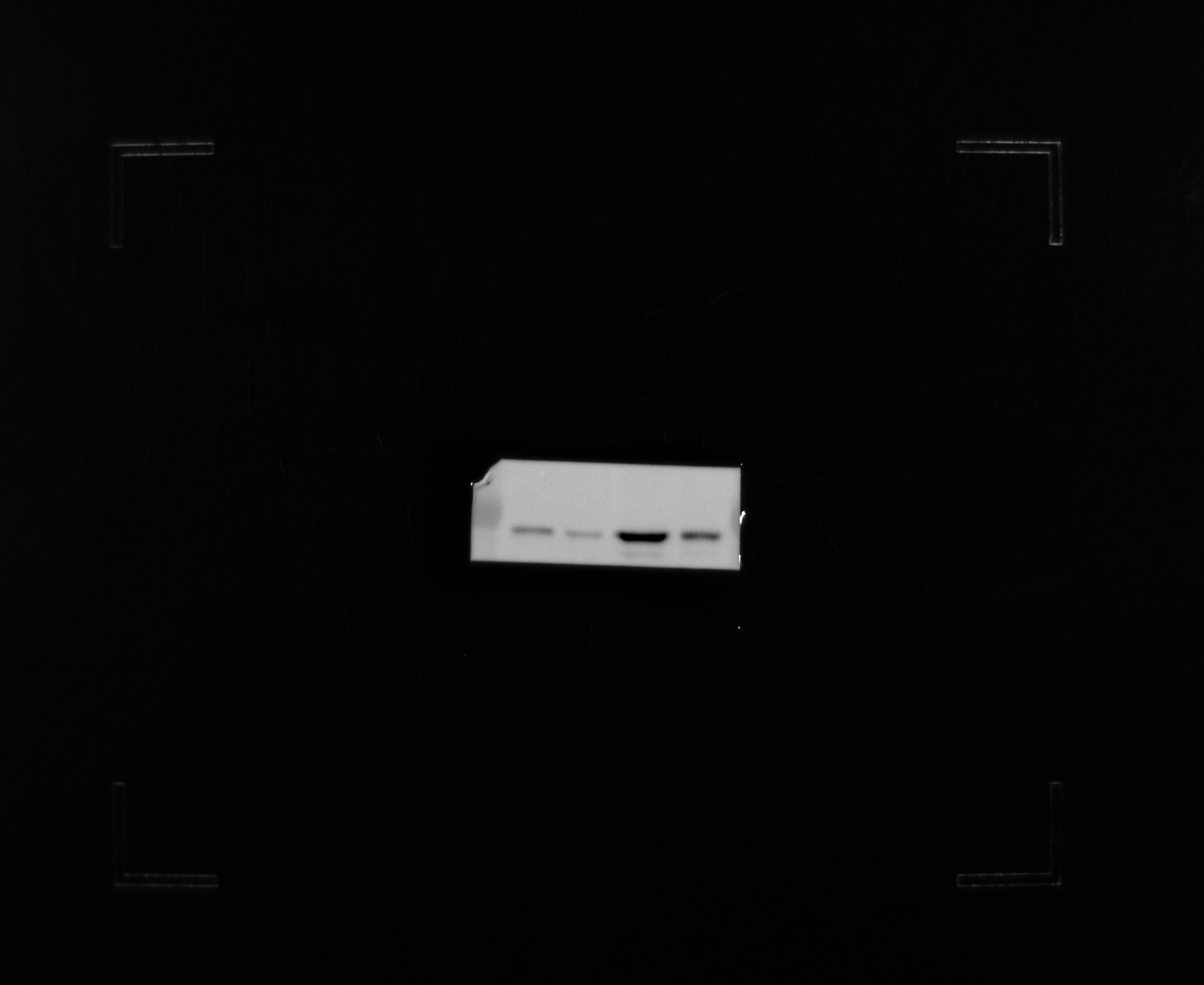

Supplement: Supplementary file 1 [file biomolecules-16-00604-s001.zip › Fig 3A-JMJD2D.tif]

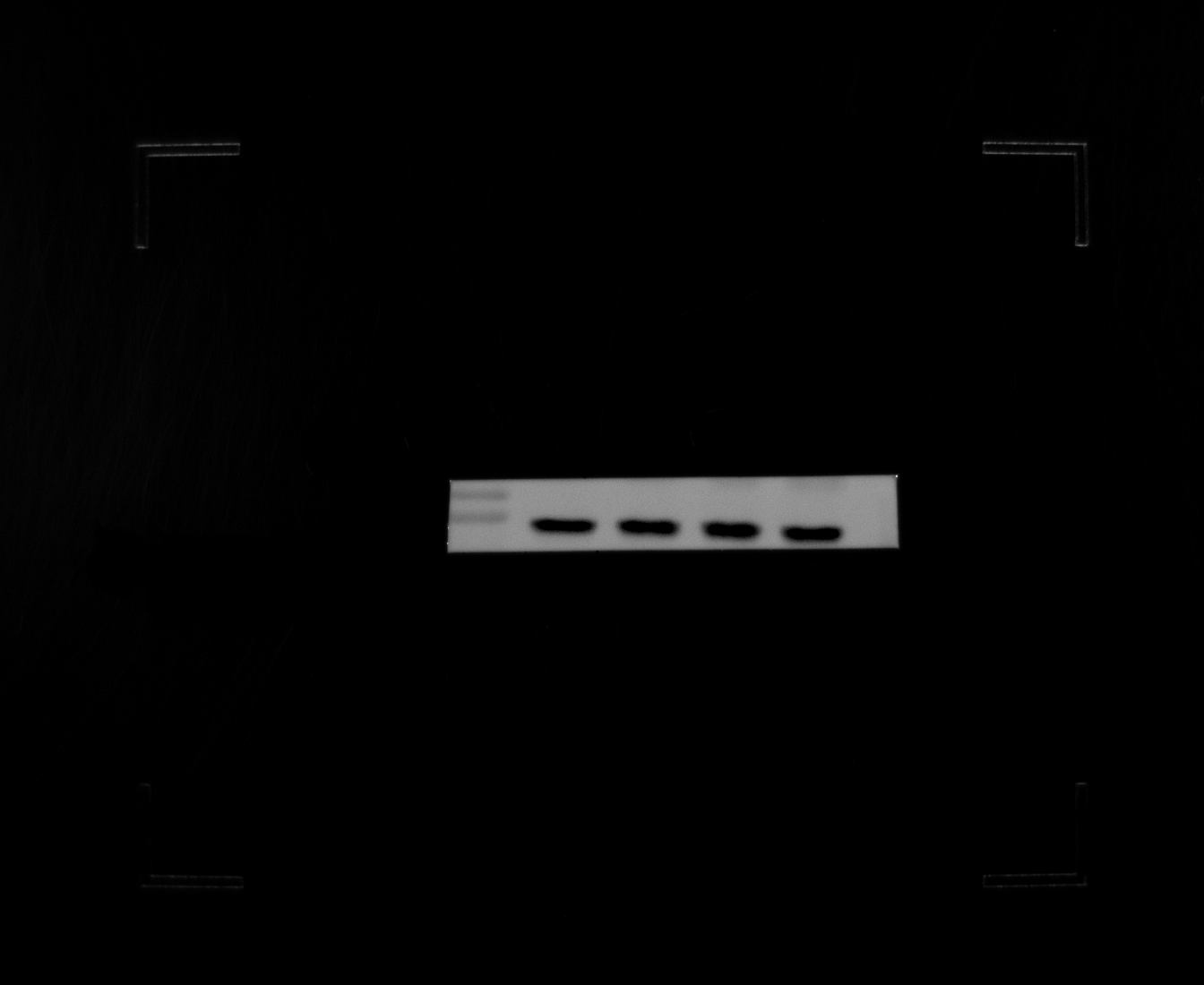

Supplement: Supplementary file 1 [file biomolecules-16-00604-s001.zip › Fig 3H-GAPDH.tif]

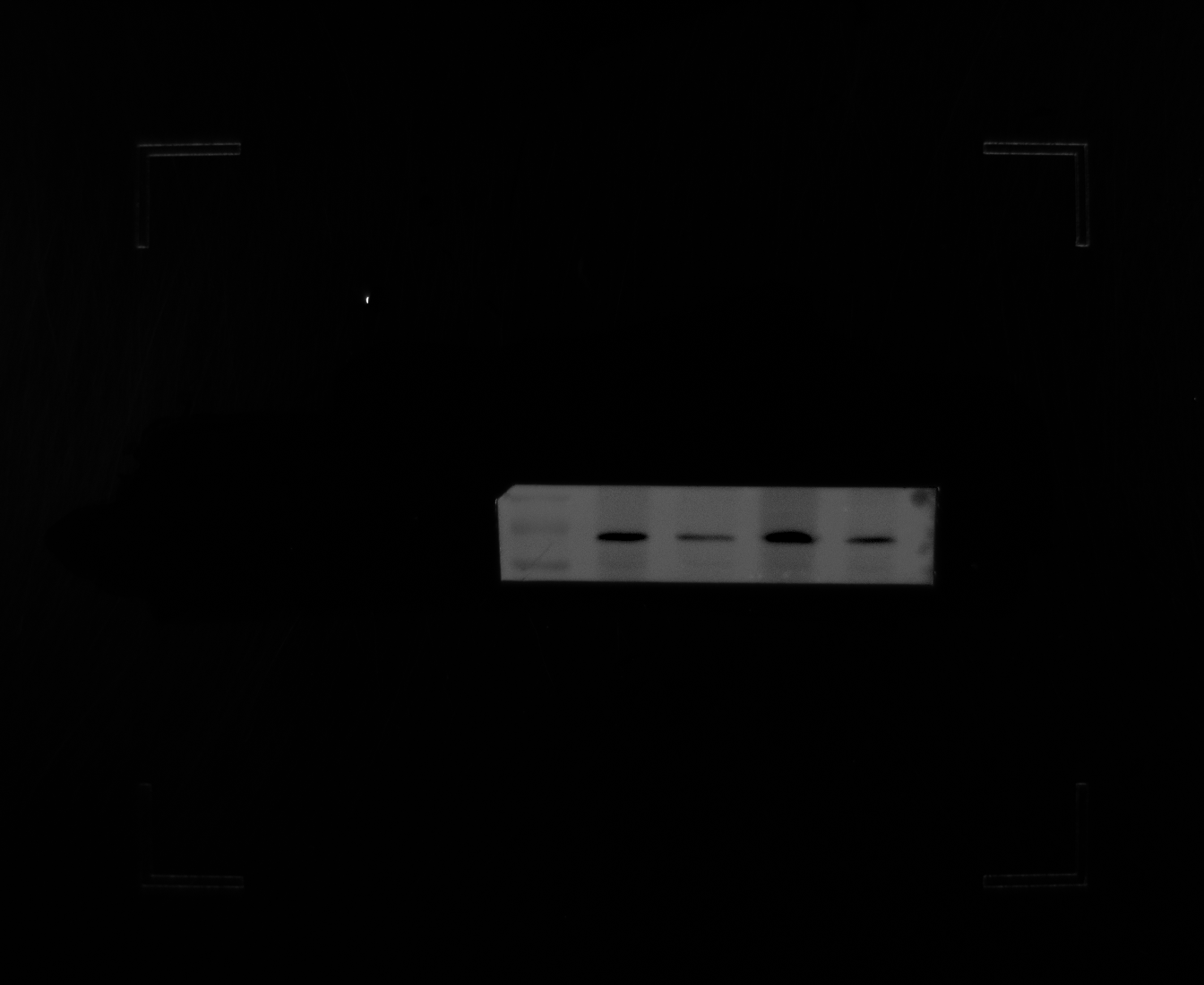

Supplement: Supplementary file 1 [file biomolecules-16-00604-s001.zip › Fig 3H-JMJD2D.tif]

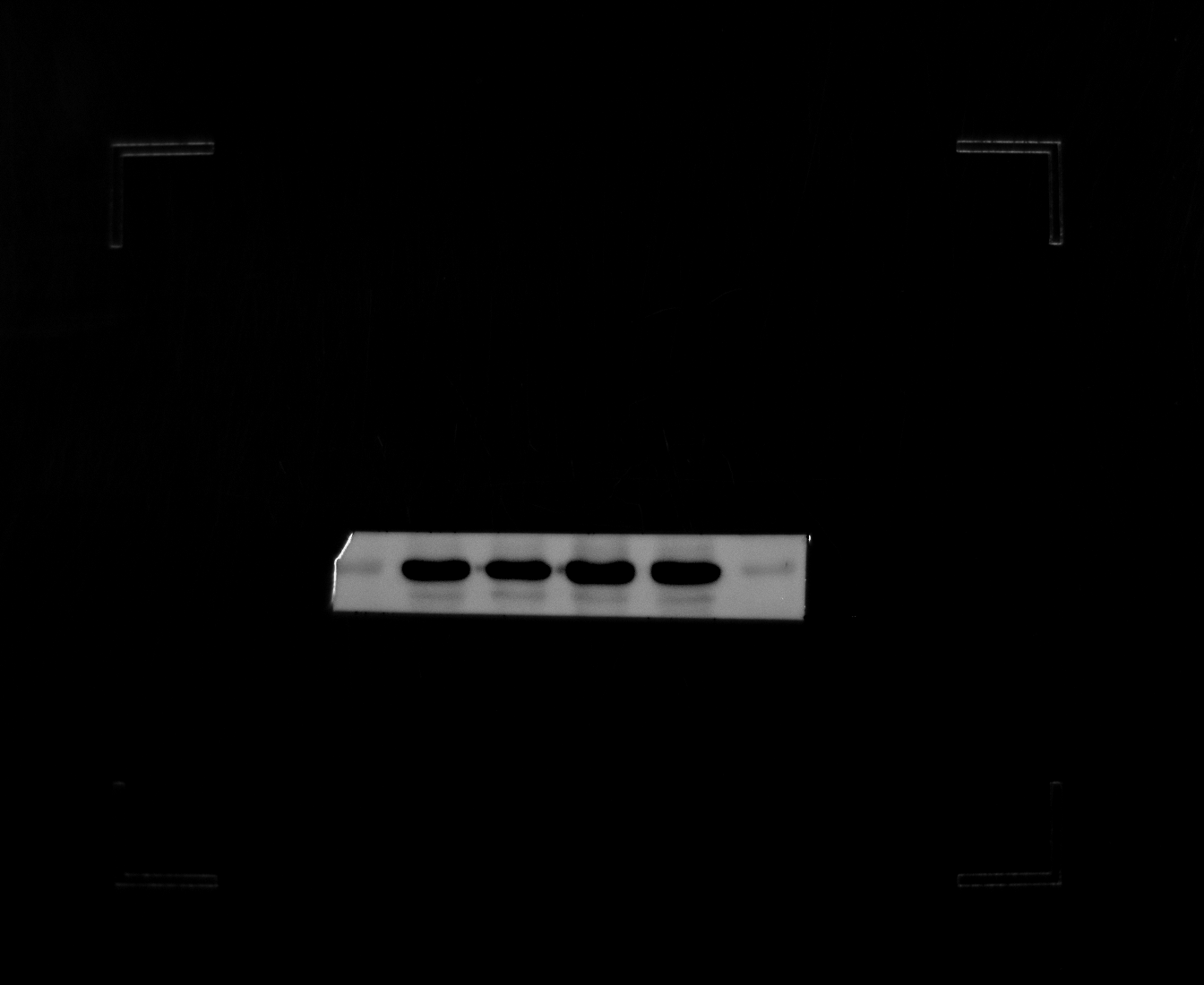

Supplement: Supplementary file 1 [file biomolecules-16-00604-s001.zip › Fig 4A-GAPDH.tif]

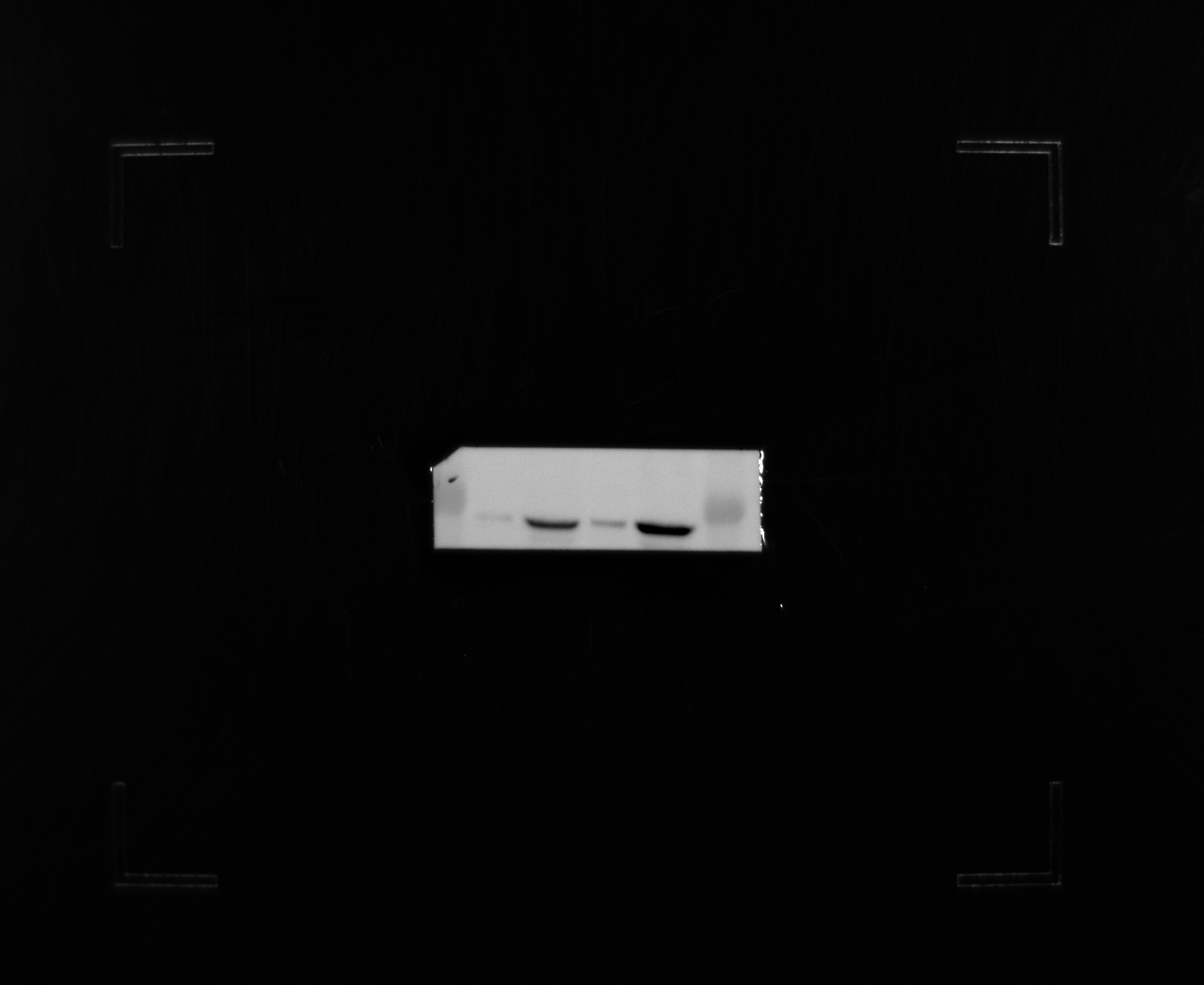

Supplement: Supplementary file 1 [file biomolecules-16-00604-s001.zip › Fig 4A-JMJD2D.tif]

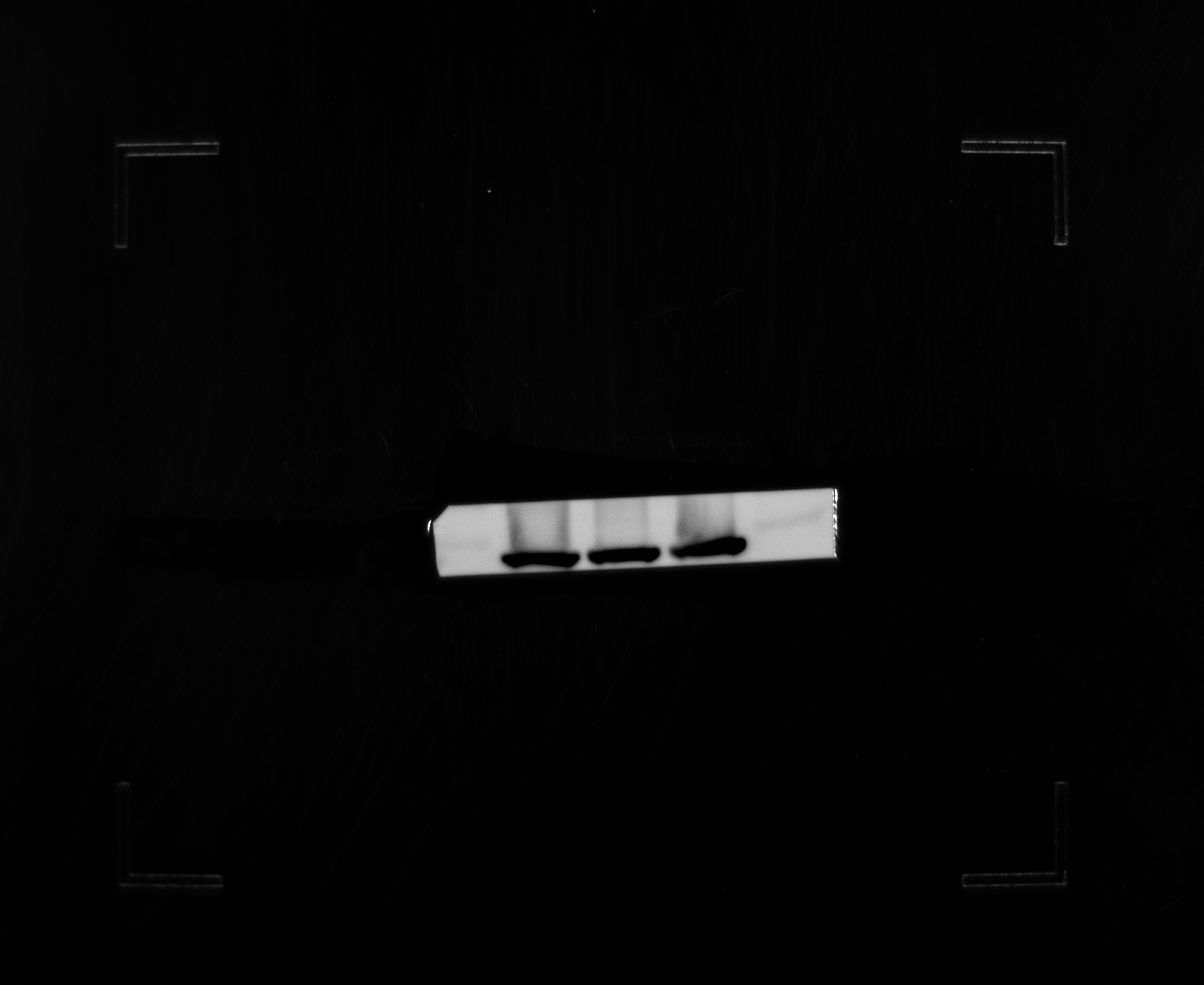

Supplement: Supplementary file 1 [file biomolecules-16-00604-s001.zip › Fig 5H-GAPDH.tif]

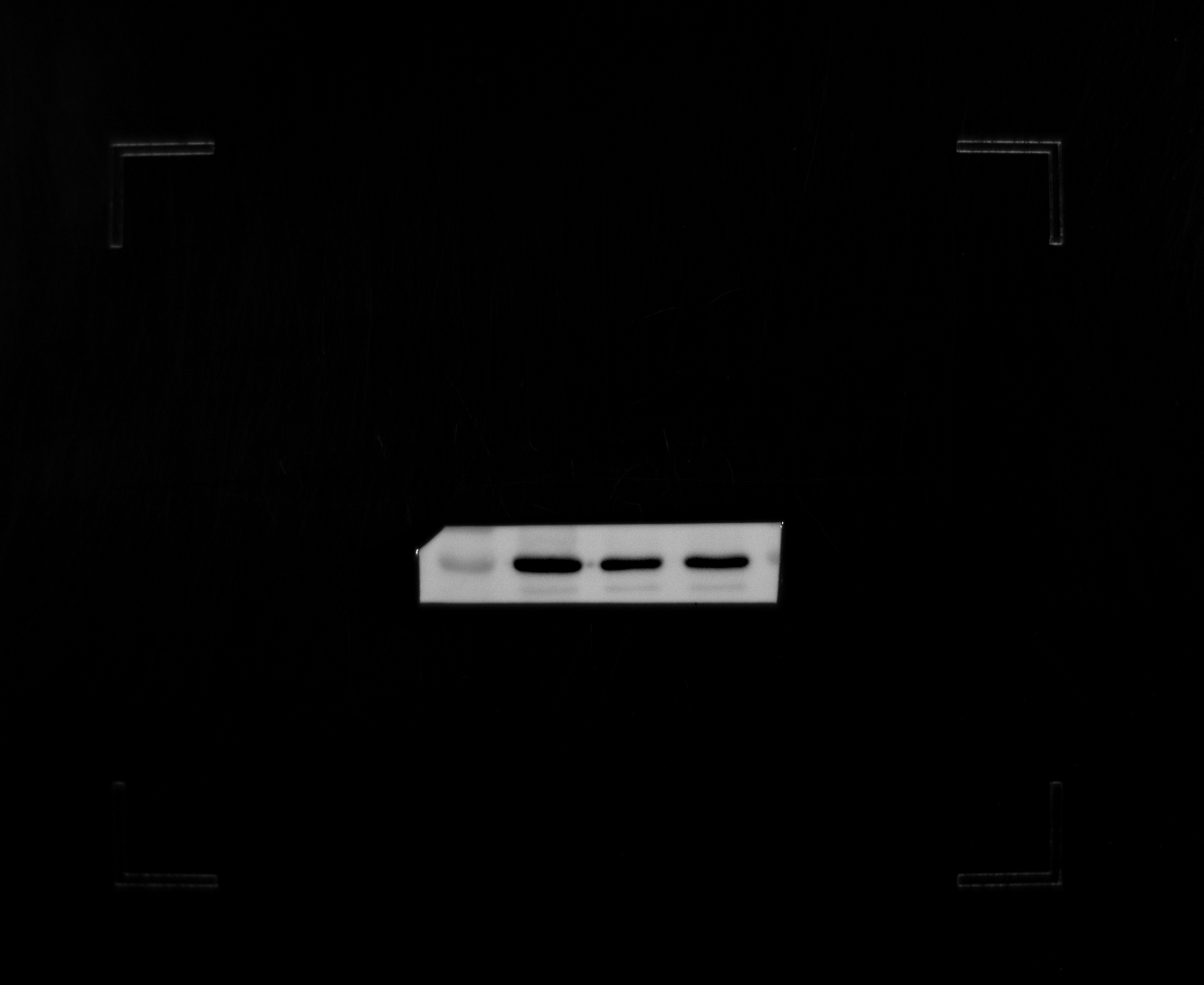

Supplement: Supplementary file 1 [file biomolecules-16-00604-s001.zip › Fig 5H-JMJD2D.tif]

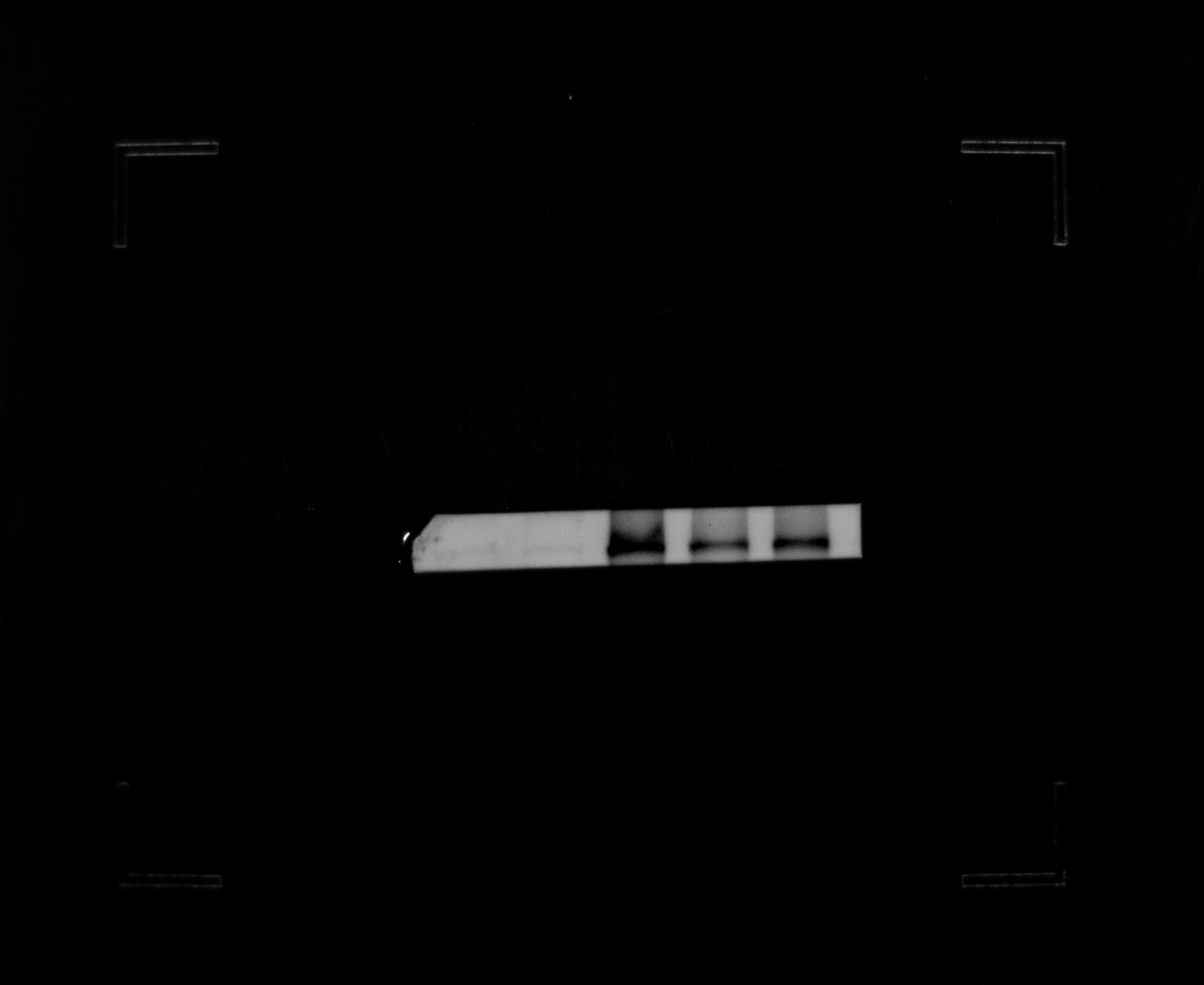

Supplement: Supplementary file 1 [file biomolecules-16-00604-s001.zip › Fig 5H-RIGI.tif]

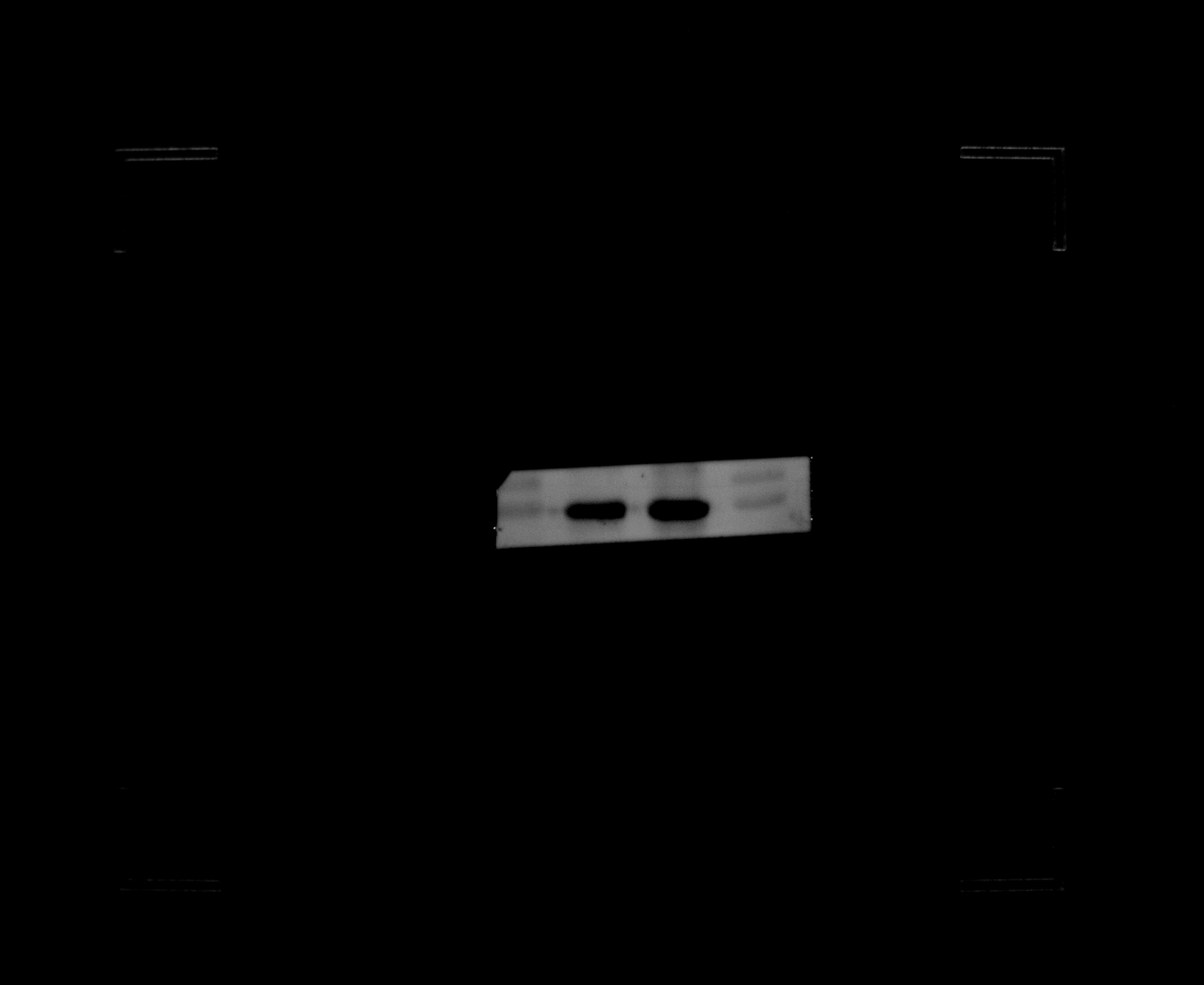

Supplement: Supplementary file 1 [file biomolecules-16-00604-s001.zip › Fig 5I-GAPDH.tif]

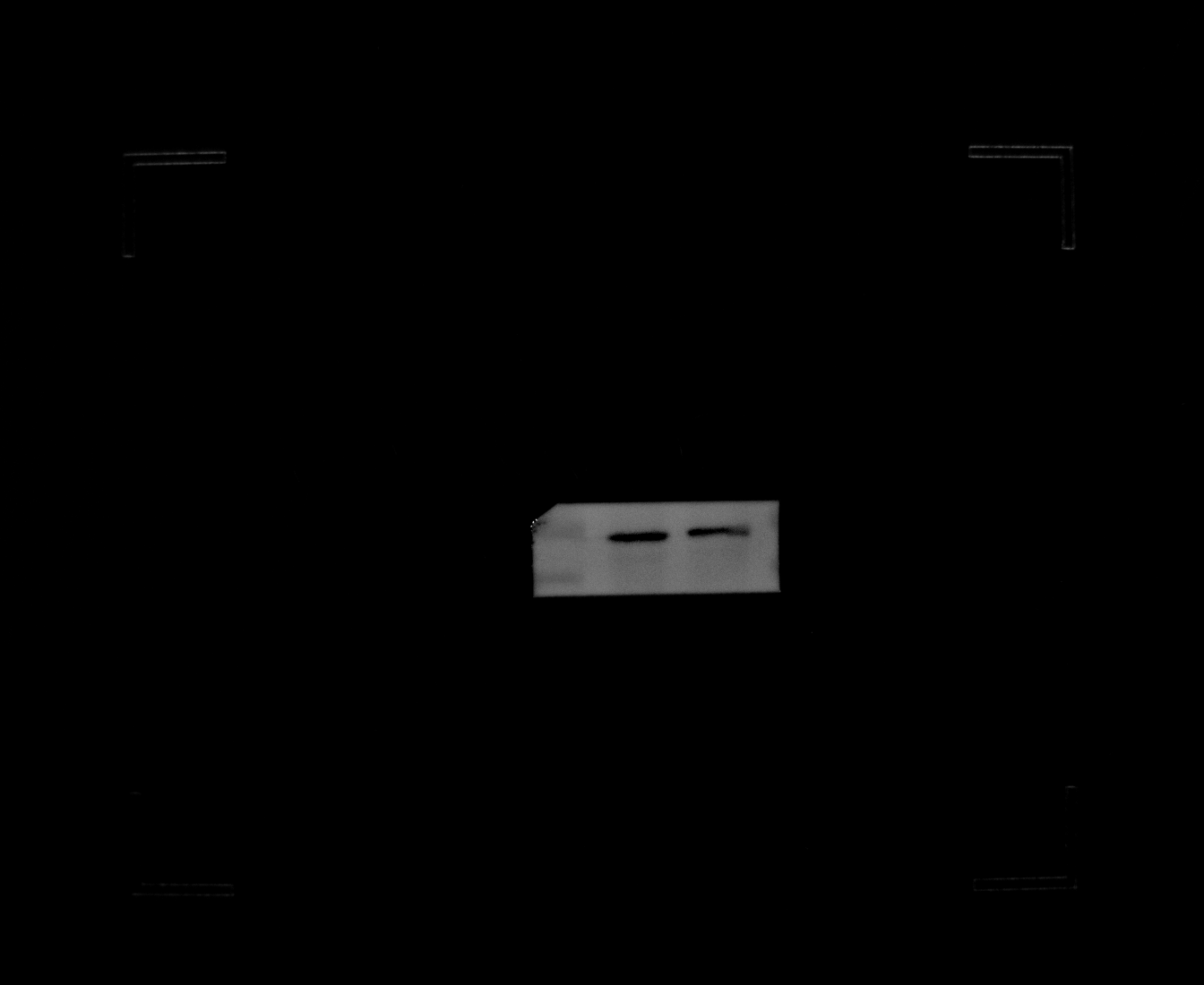

Supplement: Supplementary file 1 [file biomolecules-16-00604-s001.zip › Fig 5I-JMJD2D.tif]

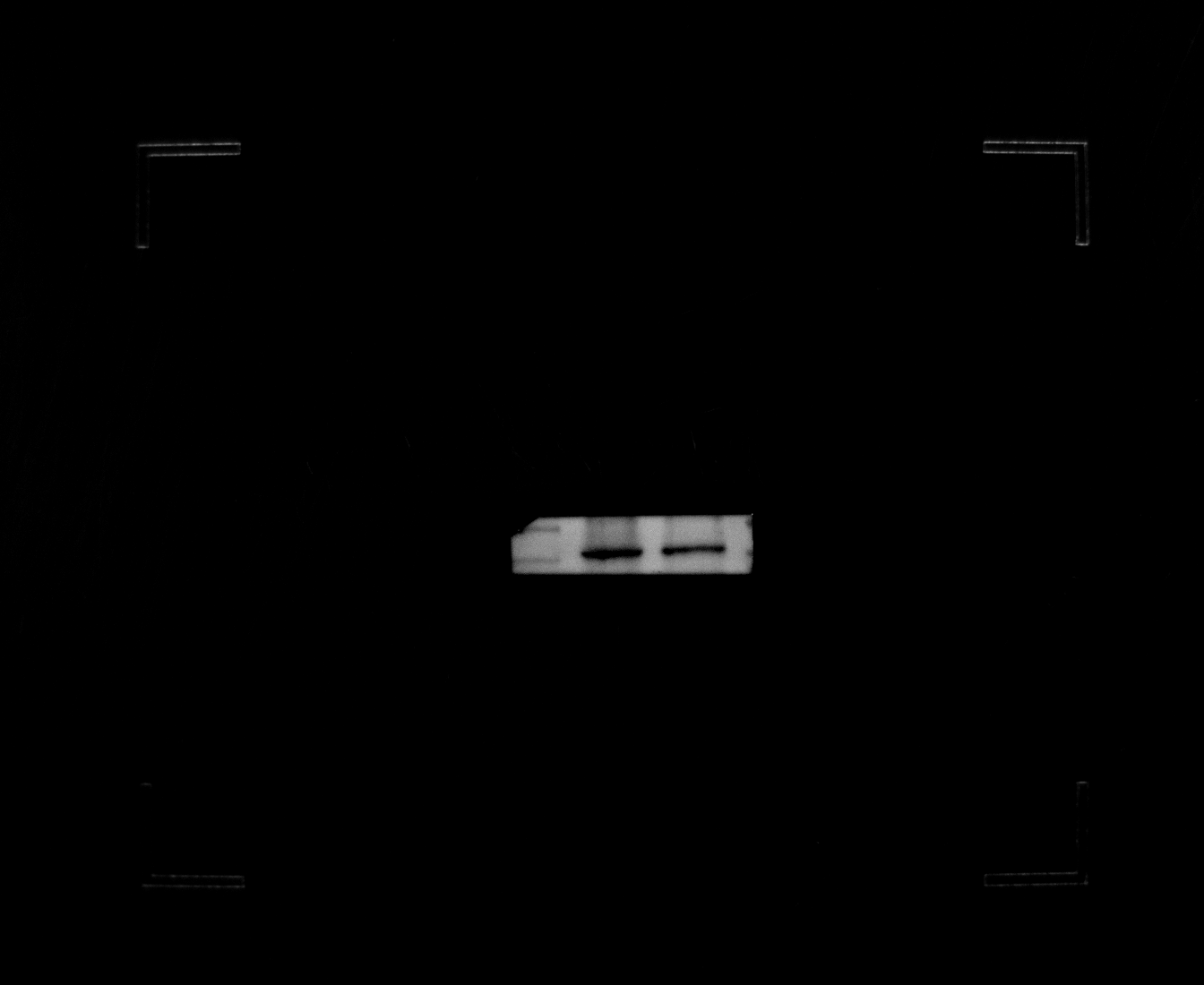

Supplement: Supplementary file 1 [file biomolecules-16-00604-s001.zip › Fig 5I-RIGI.tif]

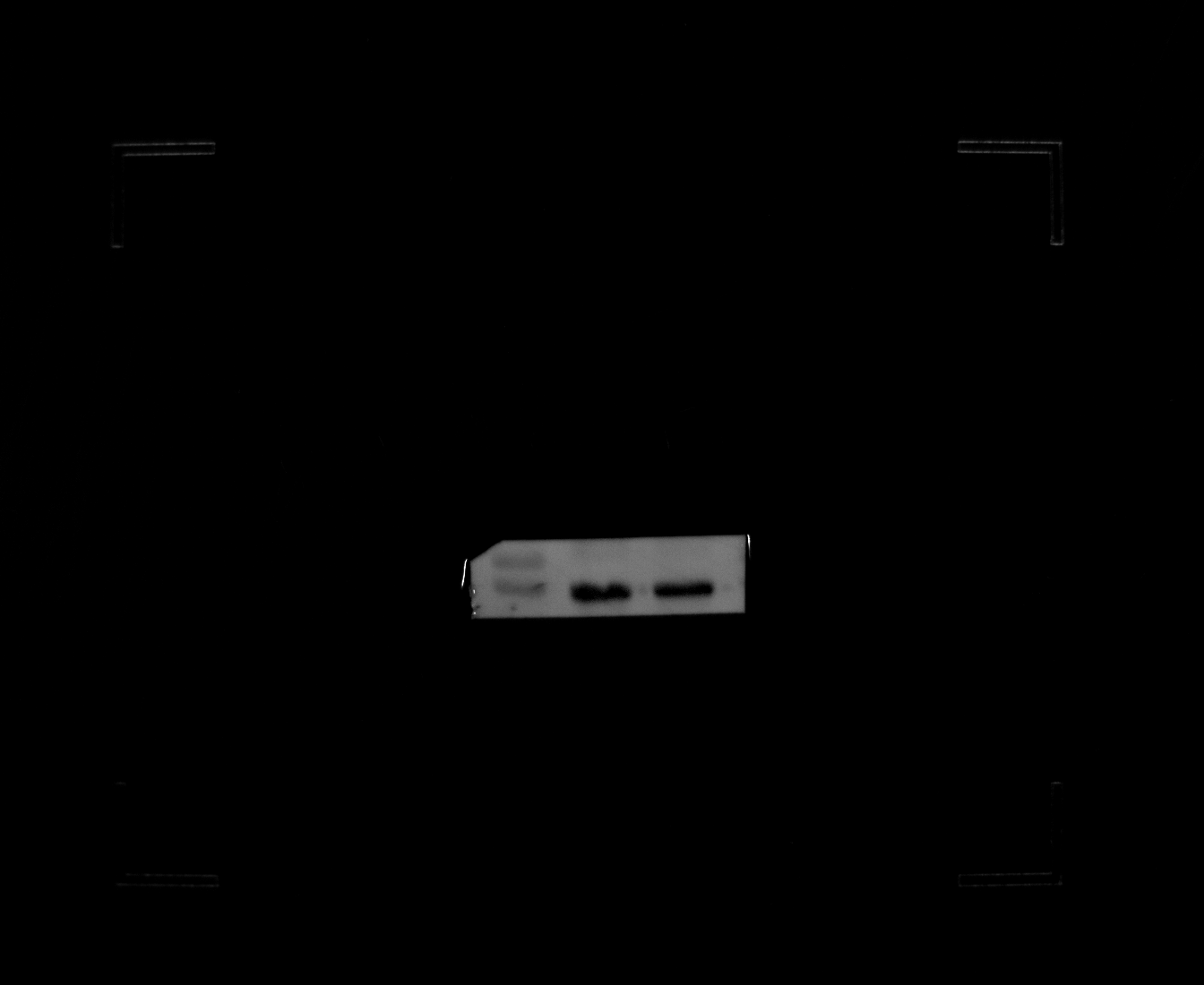

Supplement: Supplementary file 1 [file biomolecules-16-00604-s001.zip › Fig 5J-GAPDH.tif]

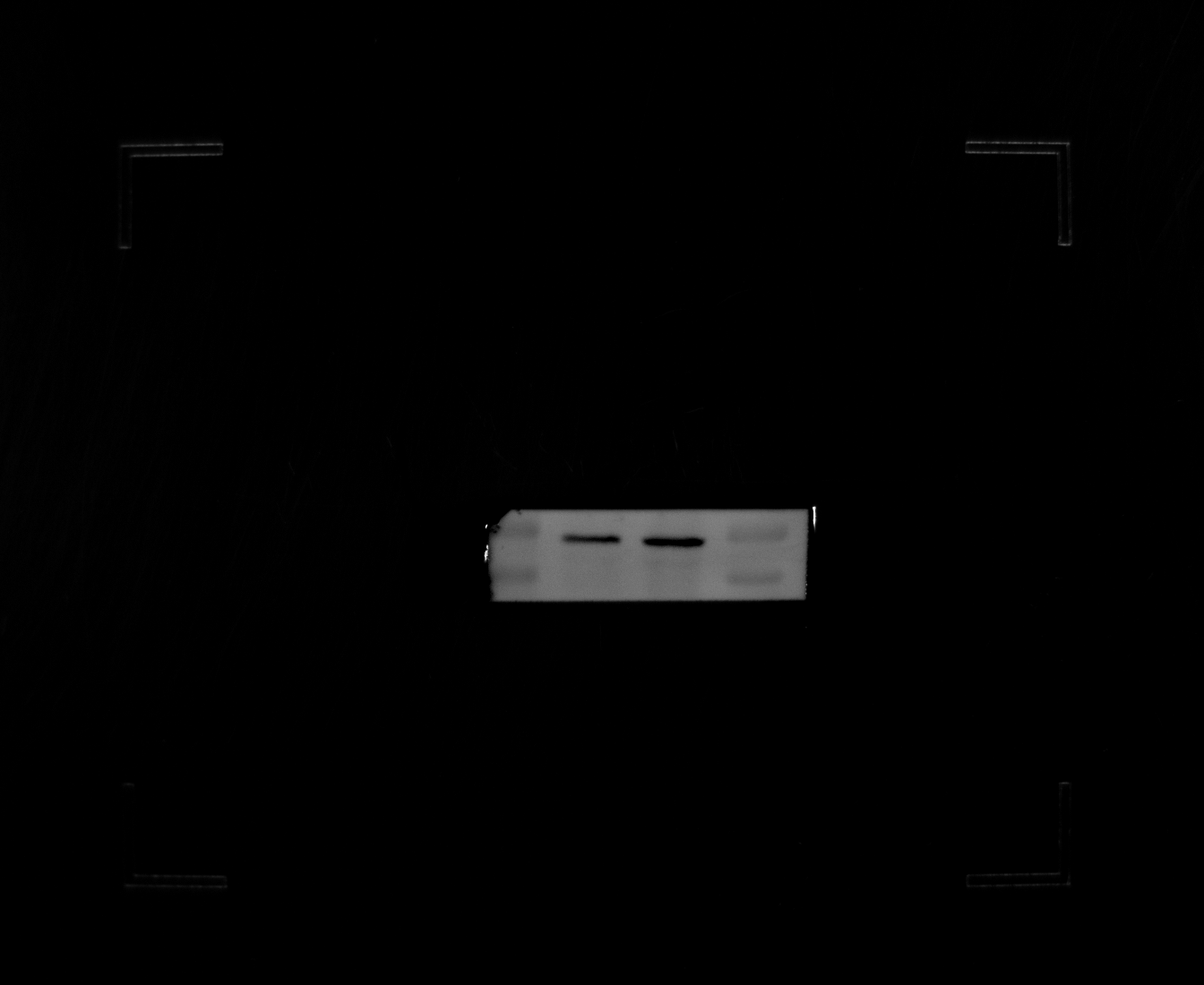

Supplement: Supplementary file 1 [file biomolecules-16-00604-s001.zip › Fig 5J-JMJD2D.tif]

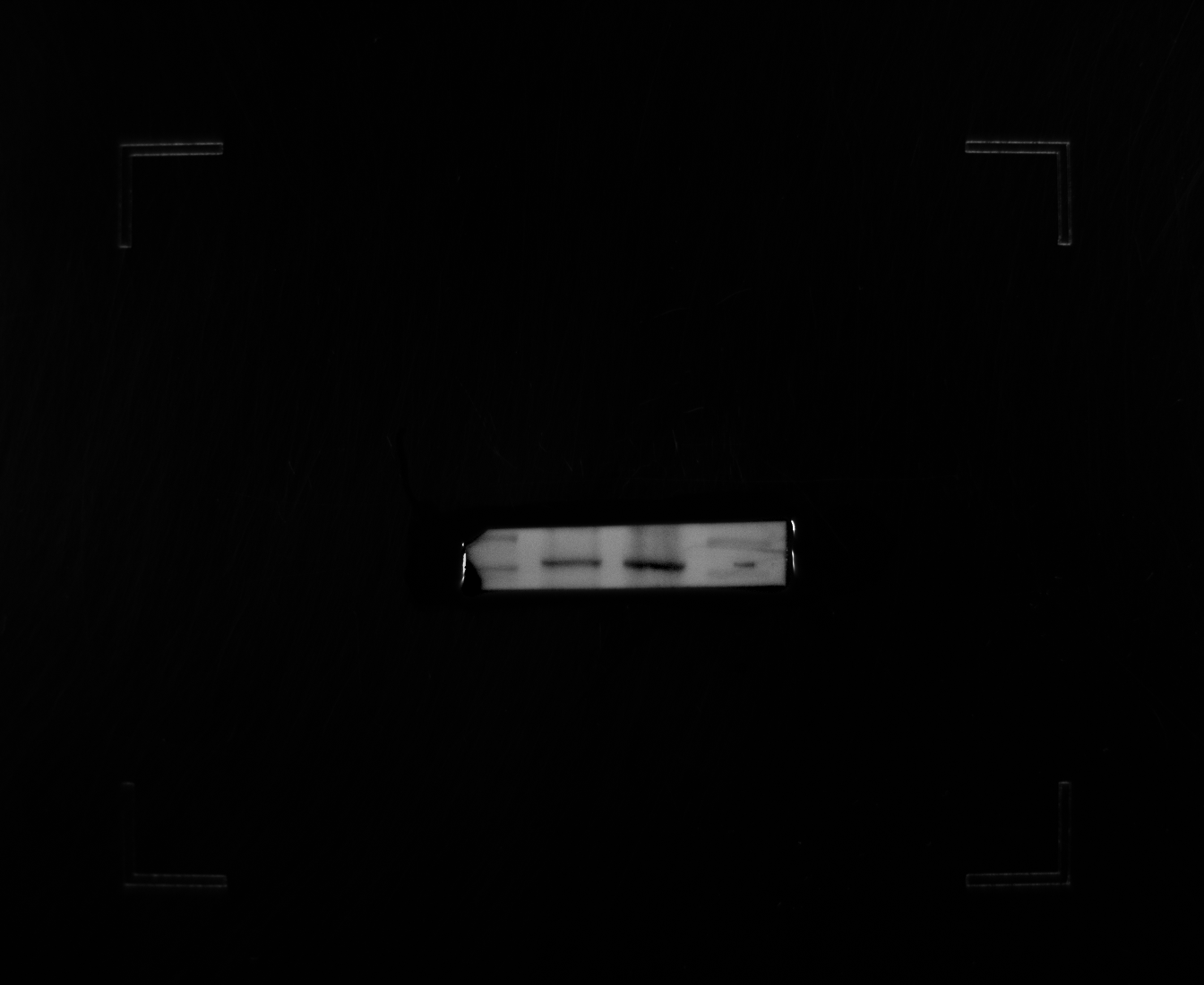

Supplement: Supplementary file 1 [file biomolecules-16-00604-s001.zip › Fig 5J-RIGI.tif]

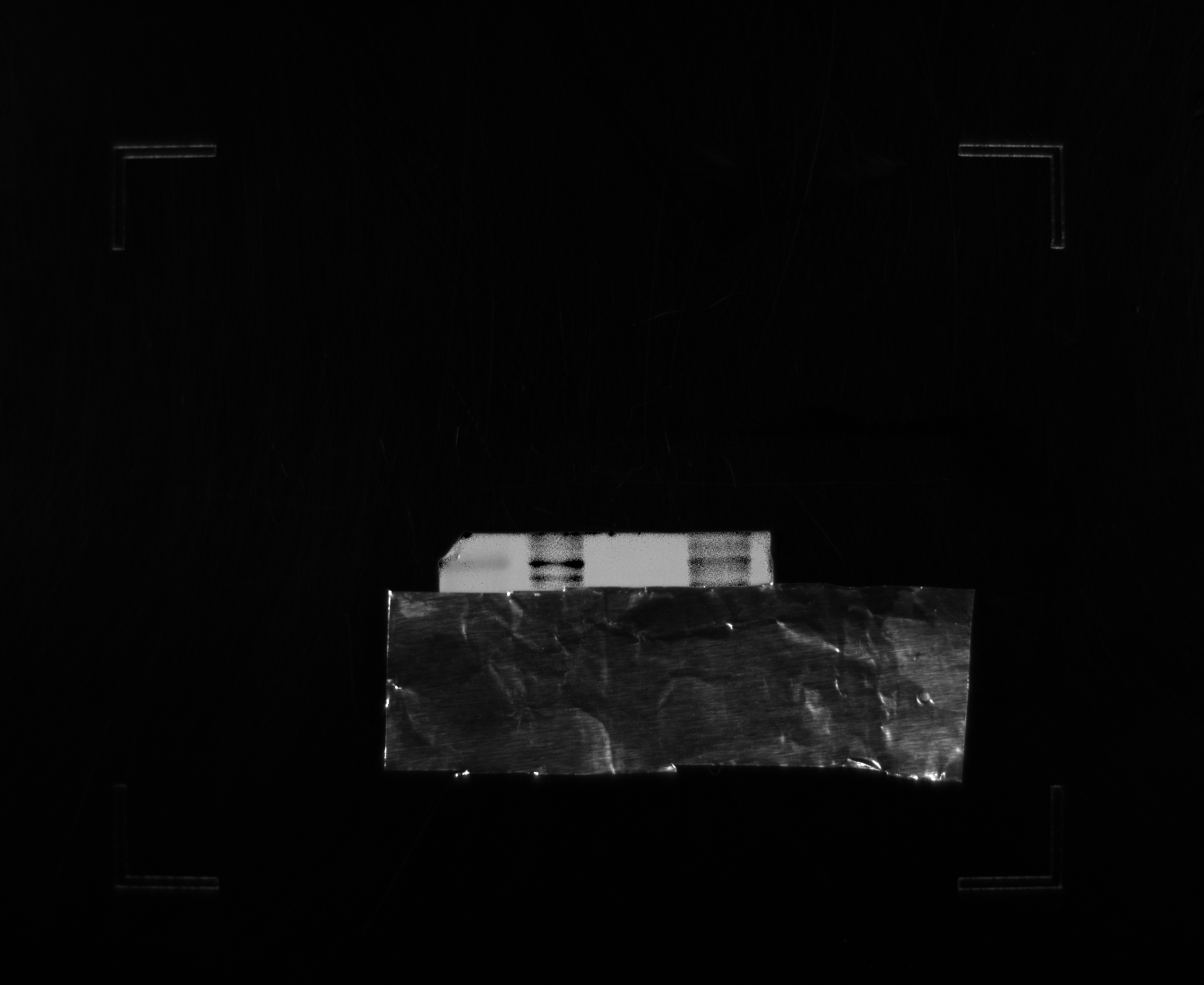

Supplement: Supplementary file 1 [file biomolecules-16-00604-s001.zip › Fig 7D-JMJD2D.tif]

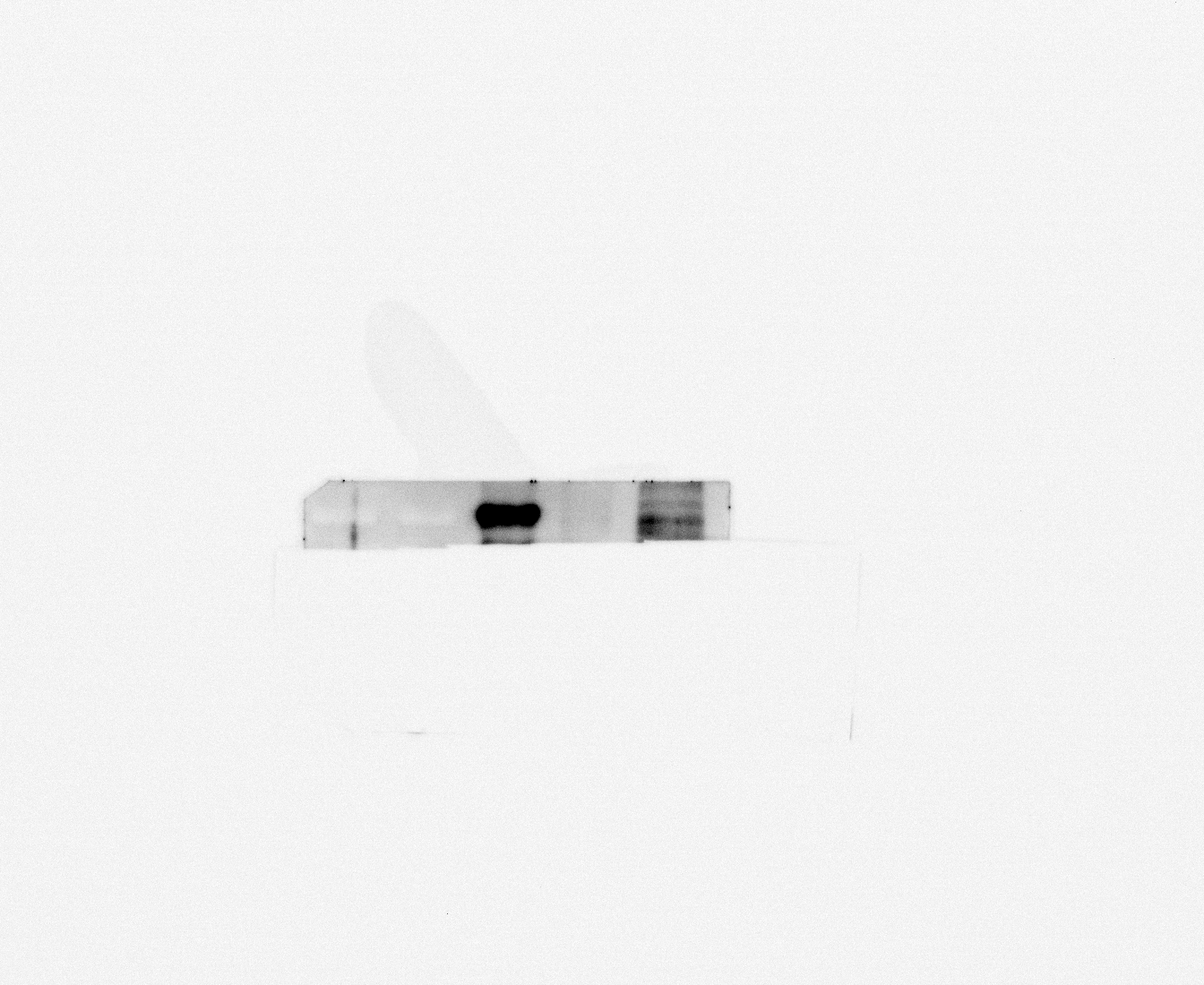

Supplement: Supplementary file 1 [file biomolecules-16-00604-s001.zip › Fig 7D-NFkB.tif]

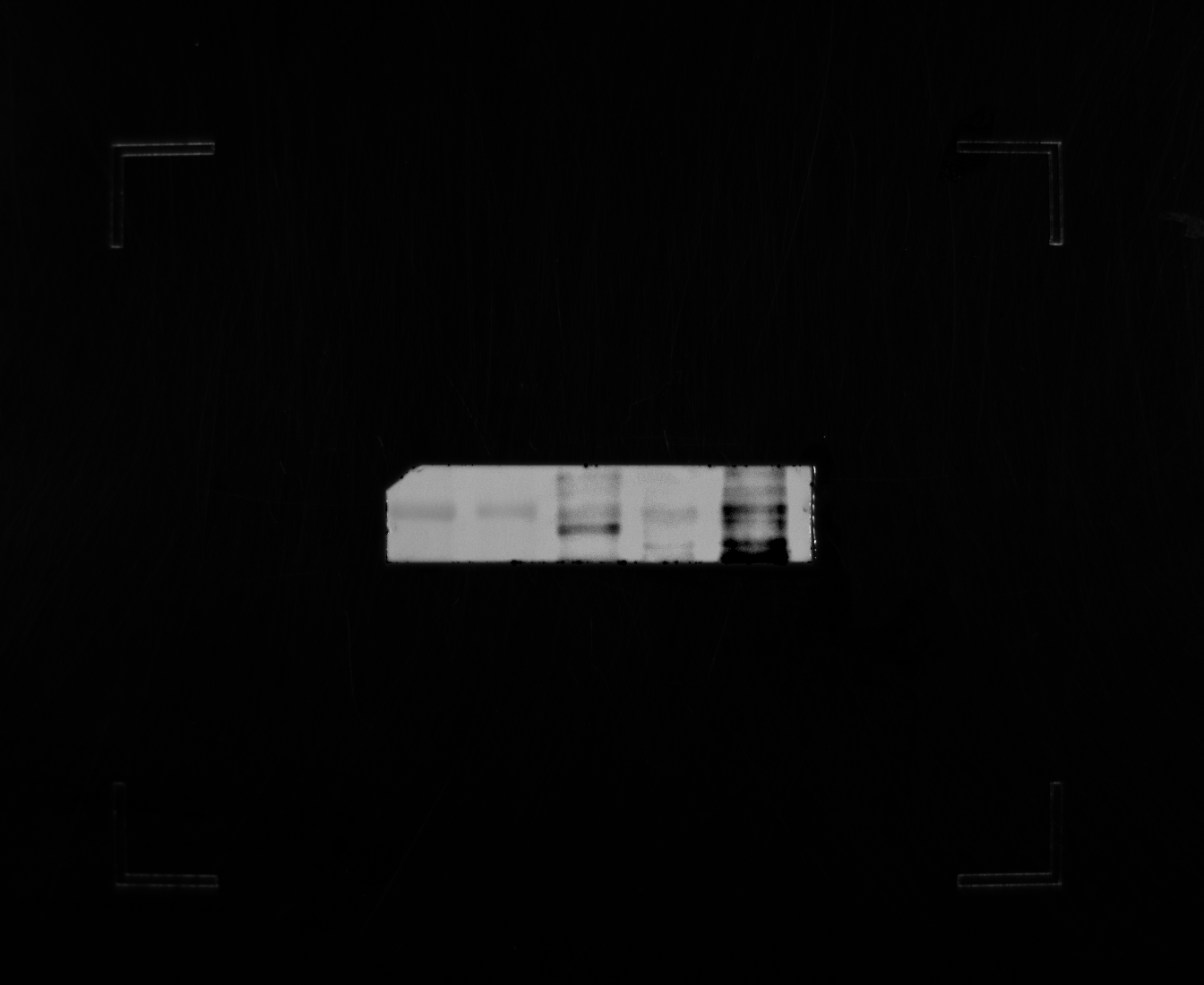

Supplement: Supplementary file 1 [file biomolecules-16-00604-s001.zip › Fig 7E-JMJD2D.tif]

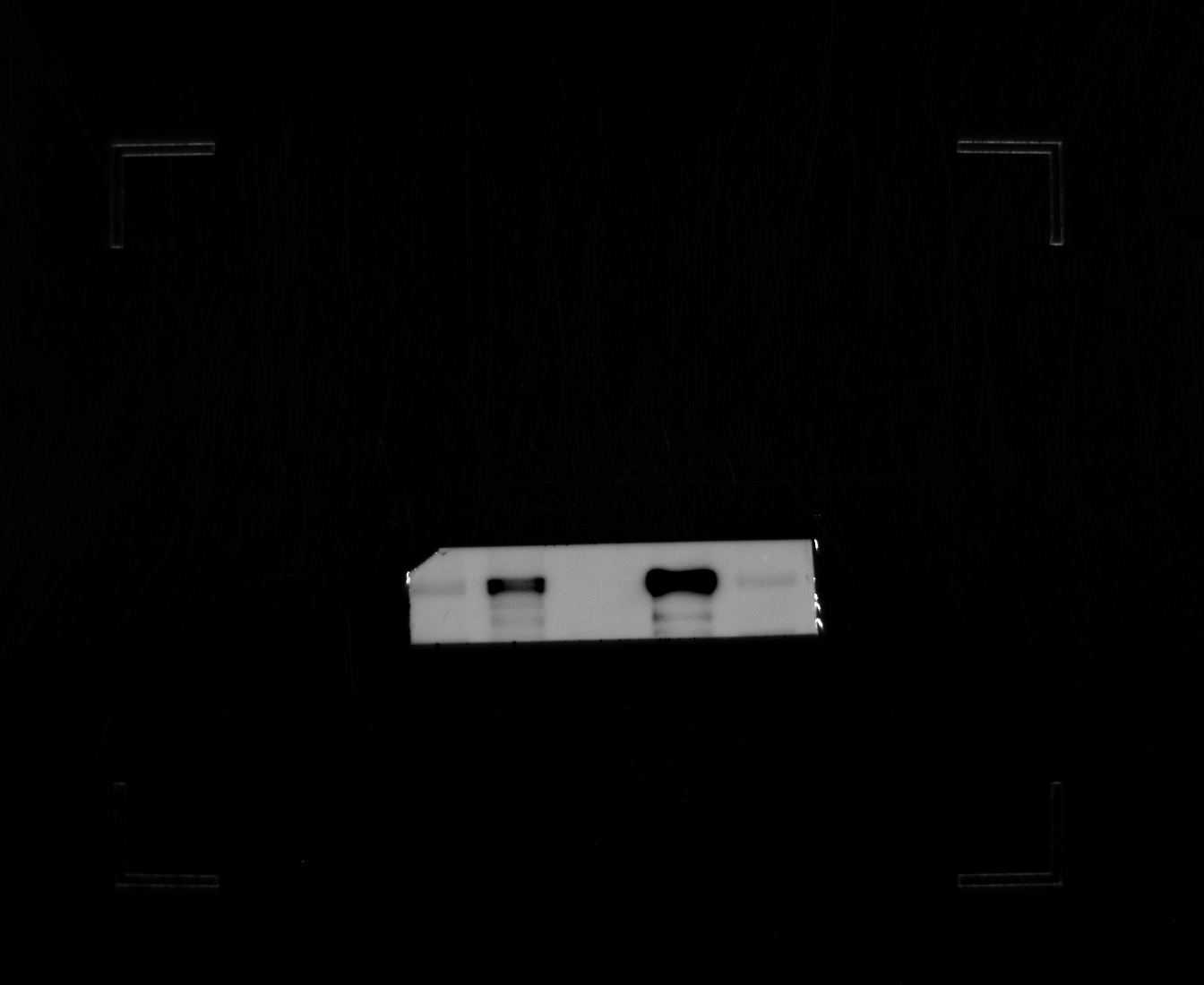

Supplement: Supplementary file 1 [file biomolecules-16-00604-s001.zip › Fig 7E-NFkB.tif]
